# Hydrostatic Pressure as a Tool for the Study of Semiconductor Properties—An Example of III–V Nitrides

**DOI:** 10.3390/ma17164022

**Published:** 2024-08-13

**Authors:** Iza Gorczyca, Tadek Suski, Piotr Perlin, Izabella Grzegory, Agata Kaminska, Grzegorz Staszczak

**Affiliations:** 1 Institute of High Pressure Physics, Polish Academy of Sciences, Sokolowska 29/37, 01-142 Warsaw, Poland; piotr@unipress.waw.pl (P.P.); izabella@unipress.waw.pl (I.G.); kaminska@ifpan.edu.pl (A.K.); staszczak@unipress.waw.pl (G.S.); 2 Institute of Physics, Polish Academy of Sciences, Aleja Lotnikow 32/46, 02-668 Warsaw, Poland; 3 Faculty of Mathematics and Natural Sciences, School of Exact Sciences, Cardinal Stefan Wyszynski University, Dewajtis 5, 01-815 Warsaw, Poland

**Keywords:** hydrostatic pressure, semiconductors, III–V nitrides, optoelectronic devices

## Abstract

Using the example of III–V nitrides crystallizing in a wurtzite structure (GaN, AlN, and InN), this review presents the special role of hydrostatic pressure in studying semiconductor properties. Starting with a brief description of high-pressure techniques for growing bulk crystals of nitride compounds, we focus on the use of hydrostatic pressure techniques in both experimental and theoretical investigations of the special properties of nitride compounds, their alloys, and quantum structures. The bandgap pressure coefficient is one of the most important parameters in semiconductor physics. Trends in its behavior in nitride structures, together with trends in pressure-induced phase transitions, are discussed in the context of the behavior of other typical semiconductors. Using InN as an example, the pressure-dependent effects typical of very narrow bandgap materials, such as conduction band filling or effective mass behavior, are described. Interesting aspects of bandgap bowing in In-containing nitride alloys, including pressure and clustering effects, are discussed. Hydrostatic pressure also plays an important role in the study of native defects and impurities, as illustrated by the example of nitride compounds and their quantum structures. Experiments and theoretical studies on this topic are reviewed. Special attention is given to hydrostatic pressure and strain effects in short periods of nitride superlattices. The explanation of the discrepancies between theory and experiment in optical emission and its pressure dependence from InN/GaN superlattices led to the well-documented conclusion that InN growth on the GaN substrate is not possible. The built-in electric field present in InGaN/GaN and AlGaN/GaN heterostructures crystallizing in a wurtzite lattice can reach several MV/cm, leading to drastic changes in the physical properties of these structures and related devices. It is shown how hydrostatic pressure modifies these effects and helps to understand their origin.

## 1. Introduction

The aim of our review is to show the very important role of high-pressure techniques in both theoretical and experimental research. We will illustrate this with an example of III–V nitrides, their alloys, and heterostructures, such as quantum wells and superlattices. We will also briefly present the related properties of laser diodes (LDs) and light-emitting diodes (LEDs). In the study of the properties of this group of semiconductors, high-pressure techniques play a particularly important role. Nitride semiconductors, such as GaN, InN, and AlN, and their solid solutions, are widely used in modern technologies due to their exceptional optical and electrical properties. They are a well-studied group of materials with great importance for applications in optoelectronics and electronics. However, on the other hand, the whole material system is still far from the level of understanding of GaAs or Si materials. In the case of quantum structures based on InAlGaN, a large number of issues are still under discussion. Pressure, as an investigative tool, helps to better understand the mechanism of light emission and the influence of carrier localization and internal electric fields. Even today, not all aspects of these phenomena are well understood. It should be noted that in the present review, we have chosen the most interesting effects associated with the application of hydrostatic pressure. Therefore, for example, we do not focus on the case of nonpolar quantum structures. Nonpolar nitride structures, grown along the *m*- or *a*-axis of the wurtzite structure, are gaining importance due to their potential advantages in terms of emission efficiency and spectral stability in optoelectronic devices. The elimination of internal electric fields leads to better optical quality and spectral stability. However, there are numerous challenges and limitations associated with their use, including challenges related to substrate availability, high price, high density of structural defects, complex fabrication processes, and ongoing technology development that limit their widespread use. In addition, there are no particularly interesting aspects of high-pressure measurements of nonpolar structures paradoxically because of the absence of an internal electric field.

Our focus is on InN, GaN, and AlN, which crystallize in the wurtzite structure. These nitrides form a subset of III–V semiconductors characterized by short bond lengths and high ionicity. Due to the wide light spectrum they cover (from 0.7 eV for InN) [1] to 6.03 eV for AlN) [2]), they have become one of the most important materials in optoelectronics, forming the basis of visible and ultraviolet (UV) LEDs and LDs. In addition, these materials have also become increasingly important in the electronics industry, enabling the design of high-power, high-voltage, and high-electron mobility transistors (HEMTs). Hydrostatic pressure is an important tool in the study of nitride materials, starting with its crucial role in their growth techniques, as they are prone to decomposition at high temperatures [3,4]. The high pressure has revealed significant differences in lattice stability between nitride compounds, confirming the high ionicity of these materials. Nitride semiconductors exhibit several interesting and atypical properties. Among these, InN is the most unique. The narrow bandgap of InN gives rise to a number of interesting physical effects. It is worth mentioning the peculiarities of InN, such as the pressure dependence of the effective mass and the effect of conduction band filling. Furthermore, the hydrostatic pressure study of the band structure of semiconductor alloys reveals interesting effects, as shown by the example of In-containing nitride alloys, such as InGaN and InAlN. It has been shown that the bandgap values depend on the geometrical arrangement of the atoms in the crystal and how it influences the bowing of the band gap and its pressure coefficient [5,6,7]. The issues presented above will be discussed in Section 2 of this review. Section 3 then describes high-pressure studies of native defects and impurities in nitride compounds. High-pressure experiments and theoretical studies have contributed to a better understanding of the physics of impurities and native defects in nitrides in particular and in all III–V semiconductors in general (e.g., GaAs doped with Si, Sn, or Ge). Regarding nitride semiconductors, special interest began in the nineties of the last century with DX states (deep states with high relaxation energy) involving oxygen in GaN [8,9] and DX-like defects of unknown origin in n-type InN [10,11]. At the same time, theoretical studies of the DX states in nitrides, including their structural aspects [12,13], have provided highly valuable information and introduced further microscopic models describing their properties. Section 4 discusses the important role of hydrostatic pressure in the study of nitride QWs and SLs. This chapter presents the role of high pressure in identifying and better understanding the mechanisms responsible for the effective emission of LEDs and LDs, which can be used for further optimization of the parameters of these optoelectronic devices. Strain effects and the related Quantum Confined Stark Effect (QCSE) [14] in quantum structures are described. It is shown how the high pressure contributed to the conclusion that growing InN on a GaN substrate is impossible [15,16,17]. In Section 5, the role of hydrostatic pressure in determining the properties of optoelectronic devices will be briefly described along with some perspectives for future research in the field of the present review. Finally, Section 6 summarizes the main points of the review.

## 2. High Pressure Effects in Nitride Compounds and Alloys

### 2.1. High Pressure in Crystal Growth

**Figure 1:**
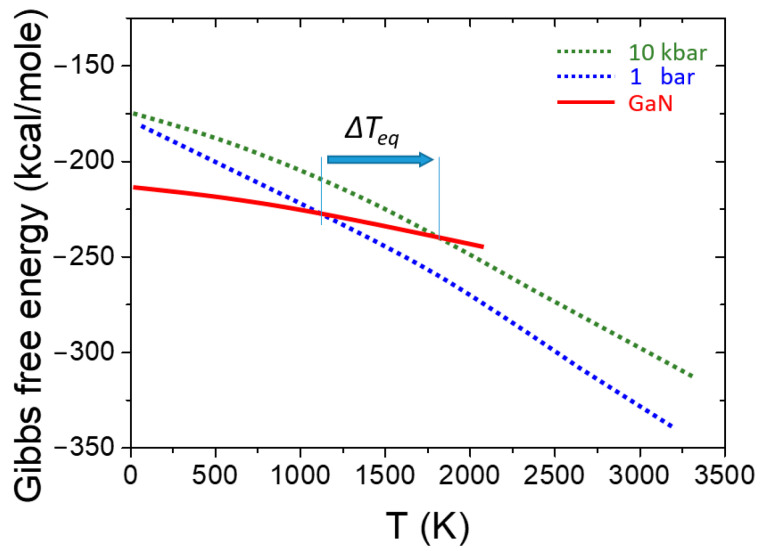
Free energy of GaN and the system of its constituents at 1 bar and 10 kbar N_2_ pressures. Adapted from Ref. [4].

High pressures can affect the physical properties of semiconductor crystals and the material systems containing them. The latter refers to pressure-induced changes in the free energies in multiphase systems, thus modifying the stability range of the system components. III-N compounds are relatively strongly bonded crystals, making them thermally and chemically stable and also inducing high melting temperatures. On the other hand, in the systems of the III-N constituents, III group metals, and N_2_ gas, an extremely strong bond in the N_2_ molecule lowers the free energy of the constituents, leading to the thermal decomposition of the III-N crystals at temperatures lower than their expected melting points. High nitrogen pressure is needed to extend the III-N’s stability range, thus suppressing their decomposition [3,4]. This situation is illustrated for GaN in Figure 1.

**Figure 2:**
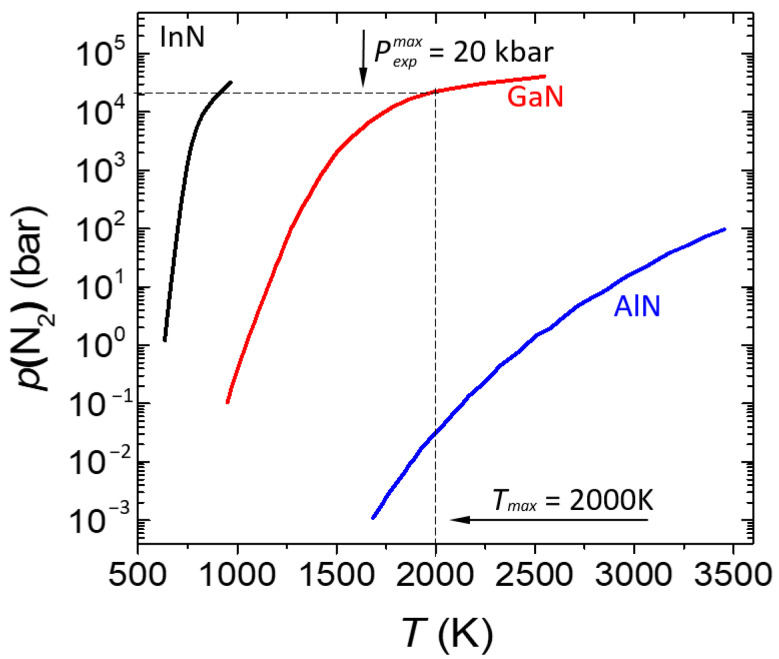
Equilibrium N_2_ pressure for the nitrides considered. The maximum pressure and temperature available in the system are indicated by dashed lines. Adapted from Ref. [4].

The extreme *p-T* conditions expected (the melting curves are still unknown!) for melting of the III-N compounds [4,18,19] make bulk crystal growth from stoichiometric melts technically unfeasible. The N_2_ pressure required for the stability of AlN, GaN, and InN at high temperatures is very sensitive to the corresponding bonding energies in the crystals. The differences in the equilibrium N_2_ pressure over III-N crystals reach several orders of magnitude, as illustrated in Figure 2.

This has direct consequences for the development of crystal growth methods for these extremely important materials:

#### 2.1.1. AlN

As it follows from Figure 2, AlN is thermally stable at atmospheric pressure [20]. A suitable method for the growth of bulk AlN is physical vapor transport (PVT), which allows the growth of up to 2-inch high-quality substrates [21,22]. However, due to the high growth temperature, the concentration of point defects is high. As an alternative, the vapor phase epitaxy (HVPE) method using ammonia as an efficient nitrogen source has been developed [23].

#### 2.1.2. GaN

It is the most suitable III-N compound for growth from solution in metallic Ga under the pressure of N_2_ gas of the order of 1.0 GPa at a temperature of the range of 1500 °C available in large-volume gas pressure reactors [3,24]. High-quality GaN crystals (dislocation density ~10^2^ cm^−2^) suitable for measurements and first homoepitaxial structures [24] were grown under conditions close to equilibrium with their constituents [3]. However, the size of the crystals was limited. Currently, the most popular methods of GaN growth are HVPE [25], ammonothermal [26], acidic [27], and Na-flux [28]. The basic method of ammonothermal growth requires the application of pressure in the range of 1 GPa. Crystals of up to 8 inches can be obtained using this method.

#### 2.1.3. InN

As already mentioned, true bulk InN crystals are not yet available. InN cannot be melted in its wurtzite phase for fundamental reasons because before reaching the melting point, the crystal decomposes or undergoes a pressure-induced structural phase transition to the cubic rock salt phase (at about 10 GPa) [19]. Only microcrystals of wurtzite InN grown by the ammonothermal method [29] and from In solution at a high pressure of N_2_ [30] were obtained. However, InN epitaxial thin films are grown by MBE instead (i.e., Ref. [31]), using nitrogen plasma as the source of active nitrogen.

### 2.2. Pressure Dependence of the Band Gaps

The energy band gaps, *E_g_*, or more generally, the electron direct (wave vector *k* is preserved) and indirect (*k* vector is not preserved) transitions between the conduction band (CB) and valence band (VB), together with their pressure dependence, are the subject of intensive studies [32] as a fundamental property of semiconductor materials. The band gaps of the typical III–V semiconductors such as GaAs, GaSb, GaP, InAs, InSb, and InP cover a relatively narrow range of values from 0.24 eV (InSb) to 2.3 eV (GaP), all of them being direct with the exception of GaP. On the other hand, the band gaps of III–V nitrides range from 0.7 eV to 6.1 eV. All are direct and together with their alloys, cover the spectrum of light from the near-infrared to UV. The bandgap pressure coefficient, *dE_g_*/*dP*, represents the shift under the pressure of the CB minimum (CBM) in relation to the VB maximum (VBM). The absolute shift of the CBM with pressure is much larger than the shift of the VBM. Therefore, it is the CBM that mainly contributes to the *dE_g_*/*dp*. The *dE_g_*/*dp*, and even its sign, is often used to identify the nature of the band gap [33]. For example, in the case of the Al*_x_*Ga_1−*x*_As alloy, the pressure coefficient *dE_g_*/*dp* changes from ~110 meV/GPa (*x* < 0.4) to ~−10 meV/GPa (*x* > 0.4) when the Al*_x_*Ga_1−*x*_As band gap becomes indirect and corresponds to the transition between VBM at the Γ point and CBM at the X point [33].

The pressure dependence of the band gap is different in nitrides and in classical III–V semiconductors. Conventional III–V compounds roughly follow Paul’s empirical rule [33] that the direct gap pressure coefficients at the Γ point are almost the same for all III–V compounds. Indeed, they typically vary between 100 and 150 meV/GPa. However, the coefficients for nitrides range from ~27 to ~50 meV/GPa. In order to explain the bandgap behavior under pressure in all III–V semiconductors, a new rule had to be formulated. It has been observed that the *dE_g_*/*dp* increases with the lattice parameter and decreases as the ionicity increases in all the compounds considered [34]. There are several scales of ionicity. In common use is the Phillips [35] ionicity scale. It remains empirical in nature since its determination involves experimental measurements. Garcia and Cohen [36] constructed a first-principles ionicity scale. Their focus was on charge density asymmetry as a measure of bond ionicity. Christensen and Gorczyca [34] obtained the ionicity values for nitrides from ab initio calculations following the procedure in Ref. [37]. Their ionicity values were very similar to those obtained by Garcia and Cohen [36]. Table 1 lists the experimental band gaps, their pressure coefficients, and lattice constants. Ionicity values from various sources are also given. The effective lattice constant for nitrides crystallizing in a wurtzite structure is given by *a_eff_* = sqrt(3)*a^2^c*, where *a* and *c* are lattice constants of the wurtzite structure.

**Figure 3:**
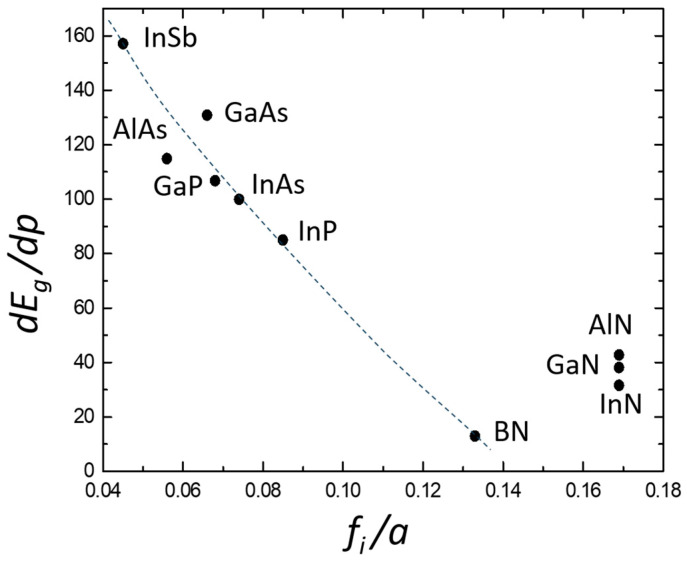
Pressure coefficients of the direct gaps of various III–V compounds as a function of the ratio between their ionicity *f_i_* and lattice constant *a*. Ionicity values are taken from Ref. [34].

In Figure 3, the *dE_g_*/*dp* values for several III–V compounds are plotted as a function of the ratio of the lattice parameter to ionicity. The calculated ionicities of nitrides are significantly higher than those of other III–V semiconductors and are more similar to those of II–VI compounds. Consequently, nitride bandgap pressure coefficients are much lower than those of typical III–V compounds. According to the type of structure in which they crystallize, the values for “classical” III–V compounds and BN are for the zinc blende structure, while the values given for AlN, GaN, and InN are for the wurtzite structure [34].

### 2.3. Bulk Modulus and Pressure-Induced Phase Transitions

Short bond lengths and high values of the bulk modulus, resulting in high hardness of materials, are typical of nitrides. The values of bulk moduli for typical III–V compounds are between 50 and 90 GPa [45], whereas the bulk moduli for nitrides are much higher, ranging from 120 to 240 GPa, depending on the source. The bulk modulus of BN is even higher (~400 GPa for the zinc blende structure) [34], but this nitride is not considered here because it does not crystallize in a wurtzite structure. Table 2 lists the values of the bulk moduli and phase transition pressures for the three nitride compounds.

**Figure 4:**
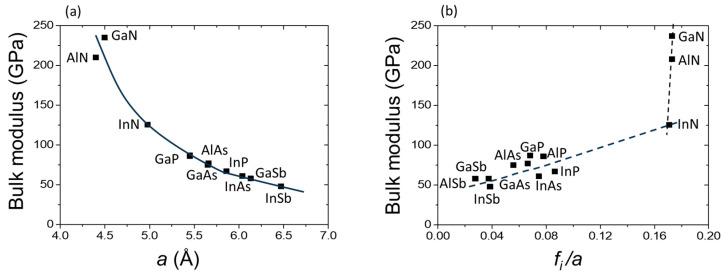
Bulk modulus for typical III–V compounds and nitrides as (**a**) function of their lattice constant, and (**b**) their lattice constant to ionicity ratio.

Figure 4a shows the dependence of the bulk moduli on the lattice parameter *a* for typical III–V semiconductors in comparison with nitrides (for nitrides, *a* = sqrt(3)*a_w_*^2^*c*, where *a_w_* and *c* are wurtzite lattice parameters). The values of the bulk moduli for nitrides (see Table 2) are taken from Refs. [46,47] (X-ray diffraction), for other compounds from Ref. [45]. The lattice constant values for nitrides are taken from Table 1 and for other compounds from Ref. [45]. We can see that *B* is inversely proportional to the lattice constant, with a strong parabolic dependence. For “classical” semiconductors, the dependence of the bulk modulus on a lattice constant is rather weak, whereas, for nitrides, it is very strong—a small change in the lattice constant results in a dramatic increase in the bulk modulus. The only problem here is the order: GaN-AlN. By tendency, AlN, which has the smallest lattice parameter *a*, should have the highest value of *B*. Instead, GaN has the highest value of *B*. A possible explanation for this discrepancy will be discussed later in this chapter.

Figure 4b plots the bulk modulus as a function of the ratio between the ionicity *f_i_* and lattice constant *a*. This Figure is analogous to Figure 3, where the band gap pressure coefficients of III–V compounds are shown as a function of the ratio between their ionicity *f_i_* and lattice parameter. Contrary to the pressure dependence of the band gaps (Figure 3), we can see that the bulk modulus increases with ionicity. It can also be observed that similar to the band gap pressure coefficients (Figure 3), the bulk modulus for nitrides is almost independent of the *f_i_*/*a* ratio. In any case, it appears that the simple dependence of *B* on the lattice constant *a* describes the behavior of the bulk modulus better than the dependence on *f_i_*/*a*. The correlation between the bulk modulus and charge density in semiconductors has been pointed out by Al-Douri et al. [45]. Using the electronic charge densities on the cation site area calculated by the empirical pseudopotential method, the values of the bulk modulus for several tetrahedrally bonded semiconductors were found to be in good agreement with the results of other authors. Under hydrostatic pressure, classical III–V semiconductors transform into the beta tin (β-Sn) structure, whereas more ionic II–VI compounds transform into the rock salt structure. The transformation of nitrides under pressure to the rock salt structure, typical of the high-pressure phase of II–VI semiconductors, is also a sign of their high ionicity. For the three considered compounds, a wurtzite to high-pressure rock salt transition has been experimentally observed without further transformations within the experimentally available pressure range. The experimental value of the transition pressure, *P_T_*, for InN is now accepted to be about 12.1 GPa [46]. It is surprising that the values of the phase transition pressure from the wurtzite structure to the rock salt structure are quite different for the two similar compounds, GaN and AlN. The transition pressure of GaN (*P_T_* = 52.2 GPa) is much higher than that of AlN (*P_T_* = 22.9 GPa), which can also be attributed to the aforementioned difference in their bulk moduli.

**Figure 5:**
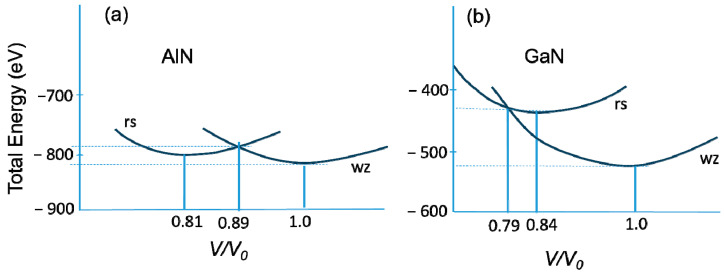
Calculated total energies for (**a**) GaN and (**b**) AlN in wurtzite (wz) and rock salt (rs) structures as functions of the relative volume *V/V*_0_.

This effect was explained by Christensen and Gorczyca [34] in 1994. It was found that the different structural stabilities could be related to the presence of *d*-like states. Ga has 3*d* states in its semi-core, whereas Al does not. Consequently, the number of unoccupied *d*-states is significantly higher in Ga than in Al, and this effect increases under pressure. This is also related to the fact that the Ga atom is “bigger” than the Al atom. This also means that the AlN crystal is “much easier” (requires less energy) to compress to the phase transition volume. This also explains why AlN has a lower value of the bulk modulus. A “flatter” slope of the total energy curves in AlN results in its lower transition pressure. The above situation is illustrated in Figure 5. For details, see Ref. [34].

### 2.4. Pressure Dependence of the Effective Mass

After the band gap and its pressure dependence, the electron-effective mass is another important parameter characterizing the band structure in semiconductors. Its values for the nitrides are given in Table 3. For the wurtzite structure, the effective mass values depend on the direction in the *k*-space. The mass associated with the *c*-axis is denoted by *m*‖, and the in-plane mass is denoted by *m*_⊥_. _⊥_The average effective mass is equal to m* = (*m*_⊥_^2^ *m*‖))^1/3^.

Special attention has been paid in the literature to the effective mass in InN and its pressure dependence. The narrow bandgap of InN (~0.7 eV) results in a strongly non-parabolic CB and one of the lowest effective masses among typical semiconducting compounds.

A deviation from the parabolicity of the CB in InN leads to differences between the optical and curvature masses. They are the same for the CBM (Γ point) but are different for other *k* values in the Brillouin zone. The effective optical mass is obtained from plasma edge absorption measurements, while the curvature mass is determined from electron transport experiments. Knowledge of their values can be very helpful in the interpretation of optical and transport experiments. Table 3 lists the experimentally obtained electron-effective masses at Γ for InN in comparison with those for GaN and AlN. The results for InN are affected by the free electron concentration and vary from 0.044 *m*_0_ to 0.07 *m*_0_.

**Figure 6:**
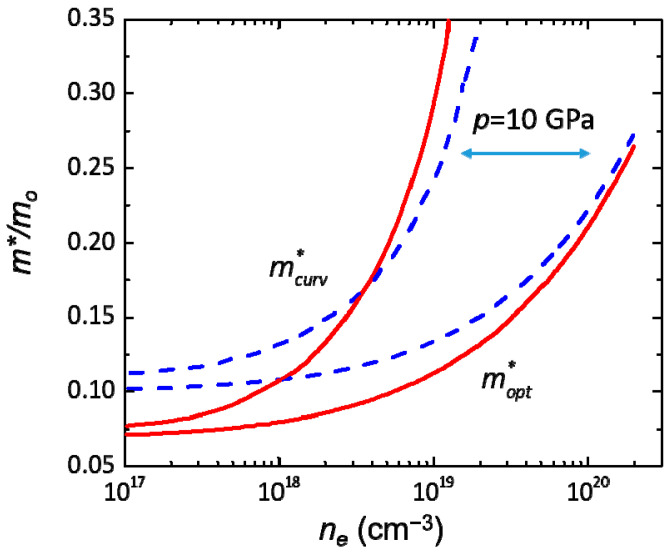
Calculated InN effective masses as a function of electron concentration for two pressures: 0 and 10 GPa. Red lines represent *p* = 0 GPa, dashed blue lines represent *p* = 10 GPa. Based on Figures 6 and 7a in Ref. [48].

In analogy to the band gap, the effective electron mass increases under hydrostatic pressure. Also, m* increases significantly with increasing electron concentration, *n_e_*. Figure 6 shows the calculated [48] InN optical and curvature effective masses as a function of the electron concentration for two pressure values: 0 and 10 GPa.

At 0 GPa, we observe a strong dependence of the curvature effective mass on the electron concentration, changing from ~0.07 *m*_0_ to ~0.35 *m*_0_ as the electron concentration increases from ~10^17^ cm^−3^ to ~10^19^ cm^−3^. For the optical effective mass, this dependence is somewhat weaker. On the other hand, the pressure coefficient, dm*/*dp*, decreases with *n_e_* due to the non-parabolic nature of the CB. In fact, as we can see in Figure 5, the dependence of m* on *n_e_* is weaker at higher pressures for both types of masses.

To experimentally verify the theoretical results above, the electron mobility was measured as a function of pressure for various values of electron concentration [48]. The obtained experimental dm*/*dp* decreases with *n_e_* from 0.056/GPa (*n_e_* = 4.2 × 10^17^) to 0.034/GPa (*n_e_* = 2.4 × 10^18^). It is important to remember that we should compare the experimental results with the electron curvature mass. However, it is observed that both masses show a similar behavior. In summary, both the calculations and the experiments show the following results:

- m* increases with electron concentration and pressure,
- dm*/*dp* decreases with increasing *n_e_*.

These effects are related to the strong non-parabolic character of the CB, which is particularly pronounced in InN.

### 2.5. Conduction Band Filling in InN

The band gap, *E_g_*, and its pressure dependence are often determined from photoluminescence, PL, measurements, assuming that *E_g_* is equal to *E_PL_*. PL is a result of the transition process from the CB to the VB. As a consequence, the PL peak energy, *E_PL_*, is equal to *E_g_* only at very low carrier concentrations when the Fermi level is not shifted up, i.e., without ‘band filling’. This means that band-filling effects must be taken into account when considering the *E_g_* or *E_PL_* behavior and its pressure dependence.

The band-filling effects are most pronounced in the narrow bandgap materials with very low m* and narrow non-parabolic dispersion of the CB; therefore, we decided to illustrate the band-filling effect using InN as an example. A theoretical and experimental investigations of the role of band-filling effects in InN were presented by Kaminska et al. [42]. The effect of CB band filling on the PL spectra was observed when a set of samples with increased *n_e_* was examined, and a blue shift of the PL spectrum was demonstrated. To compare the theory and experiment, the *E_PL_* was identified with *E_opt_*. This was justified by the assumption that at the low temperatures at which the PL measurements were carried out, recombination occurred between the Fermi levels of the CB and VB, and in this case, *E_PL_* = *E_opt_* = *E_g_* + *E_F_*, where *E_F_* is the Fermi level position.

**Figure 7:**
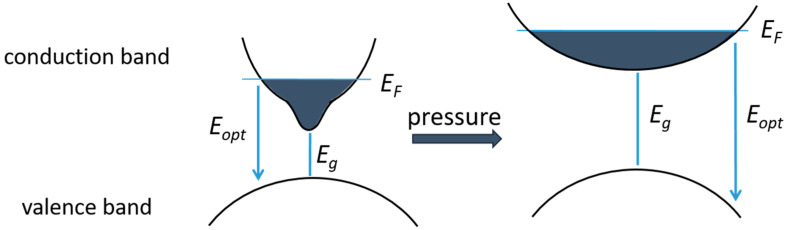
Effect of pressure on the optical band gap *E_opt_* at high-electron concentration. Black areas cover identical areas, as the number of electrons does not vary with pressure. Based on Figure 4 in Ref. [42].

PL measurements were performed under hydrostatic pressure for several samples with increasing electron concentrations. Applying pressure increases the band gap, resulting in a weaker interaction between the CB and VB and, thus, a flatter CB. The influence of pressure on the VB is much smaller and can be neglected. Therefore, assuming that the number of electrons does not change with the applied pressure, the *E_F_* is expected to decrease with increasing pressure, which is schematically illustrated in Figure 7. It can be observed that, due to the decreasing *dE_F_*/*dp*, the pressure coefficient *dE_PL_*/*dp* also decreases with increasing electron concentration, which can be expressed explicitly by the equation:

*dE*_*PL*_/*dp* = *dE*_*opt*_/*dp* = *dE*_*g*_/*dp* + *dE*_*F*_/*dp* (1)

A significant band-filling effect in InN has serious consequences because the decrease in the *dE_opt_*/*dp* with respect to *dE_g_*/*dp* makes it more difficult to identify the true value of *dE_g_*/*dp*. The effect of band-filling must be taken into account for the proper interpretation of the *dE_PL_*/*dp* values obtained from the PL measurements under pressure. It is especially important in the narrow band gap semiconductors with a low m* value.

The influence of the CB filling in InN on the PL spectra under pressure was also investigated by Franssen et al. [54]. They found that the *dE_PL_*/*dp* of InN changed from ~27 to ~21 meV/GPa as *n_e_* increased from 3.6 × 10^17^ cm^−3^ to 1.1 × 10^19^ cm^−3^. No significant change in *dE_PL_*/*dp* with *n_e_* was observed for the In_0.7_Ga_0.3_N alloy in the same study. This could be caused by a reduced pressure sensitivity of m* due to the larger band gap of the alloy.

### 2.6. Pressure Dependence of Band Gaps in In-Containing Nitride Semiconductors

The bandgap pressure coefficients, *dE_g_*/*dp*, of In-containing alloys have been experimentally determined in many papers, e.g., Refs. [5,7,54,55] for InGaN and Refs. [6,55,56] for InAlN. Interesting, however, are the *E_g_* and *dE_g_*/*dp* bowings. Comparing their values in nitride alloys, it can be seen that for alloys containing In: InGaN and InAlN, particularly large bowings are observed, while in AlGaN, these effects are less pronounced and similar to those observed in most semiconductors. Also, no clustering effects are observed in AlGaN when considering the composition dependence of the bandgap. This suggests that all these effects are related to the specific role of indium. In-containing alloys, InGaN and InAlN, are nitride alloys that are very useful in various applications due to the special role of In. Even a small amount of In in an alloy will lead to an increase in the intensity of light emission in optoelectronic devices. In*_x_*Al*_1−x_*N, which has a very wide range of *E_g_*, is a strong candidate for optoelectronic applications that operate over the light spectrum from deep UV to far infrared.

**Figure 8:**
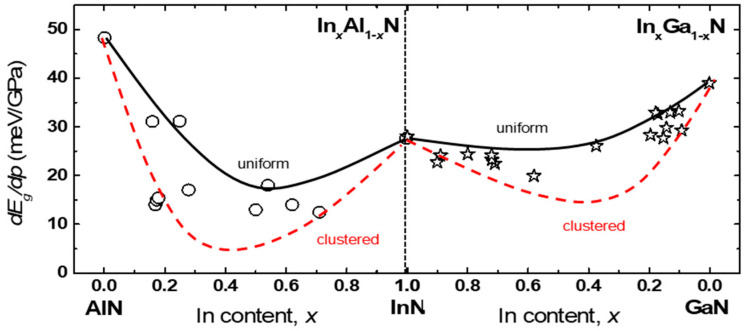
*dE_g_*/*dp* as a function of *x* in In*_x_*Al_1−*x*_N and In*_x_*Ga_1−*x*_N. Symbols represent the experimental results. Solid and dashed lines represent the uniform and clustered arrangements of indium atoms, respectively. The experimental results for In*_x_*Ga_1−*x*_N (stars) are obtained from Ref. [5] and for In*_x_*Al_1−*x*_N (circles) from Refs. [6,56].

Both experimental and theoretical methods have been used to study the electronic band structures of In*_x_*Al_1−*x*_N and In*_x_*Ga_1−*x*_N alloys with and without hydrostatic pressure. The results showed that the band gaps and their pressure coefficients, *dE_g_*/*dp*, exhibit significant bowing as a function of *x*. Ab initio calculations revealed a strong enhancement of this effect in the case of clustered distributions of In atoms [5,6,7]. PL measurements were performed to verify the theoretical dependence of *E_g_* and *dE_g_*/*dp* on the indium concentration in In*_x_*Ga_1−*x*_N and In*_x_*Al_1−*x*_N alloys, assuming that *E_PL_* corresponds to the band-to-band radiative transition. In this review, we focus only on the pressure effects. Figure 8 compares the calculated and measured *dE_PL_*/*dp* and *dE_g_*/*dp* as functions of *x* for In*_x_*Ga_1−*x*_N [5,7] and In*_x_*Al_1−*x*_N [6,56]. The solid lines represent a uniform distribution, while the dashed lines represent a clustered distribution of indium atoms. The theoretical *dE_g_*/*dp* in In*_x_*Ga*_1−x_*N decreases as *x* increases, starting from a value of *dE_g_*/*dp* = 40.6 meV/GPa for GaN. The value of *dE_g_*/*dp* in InN reaches a minimum of 27.8 meV/GPa. In In*_x_*Al_1−*x*_N, this value decreases with increasing *x*, starting from 48.5 meV/GPa for AlN until it reaches a minimum of 25 meV/GPa close to *x* = 0.5 (uniform atomic arrangement) or 16 meV/GPa close to *x* = 0.3 (clustered atomic arrangement). Figure 8 shows that the theoretical results demonstrate a more significant bowing of *dE_g_*/*dp* in In*_x_*Al_1−*x*_N compared to In*_x_*Ga_1−*x*_N, particularly in the clustered case. The theoretical values of dE_g_/dp are compared with the experimental data of *dE_PL_*/*dp* for In*_x_*Ga_1−*x*_N from Ref. [5] and for In*_x_*Al_1−*x*_N from Refs. [6,56].

The scatter of the experimental values can be an indication of different degrees of indium clustering due to different growing conditions. This evidence suggests the existence of short-range In-cation clustering, which causes changes in the electronic band structure. The measurement of *dE_PL_*/*dp* can serve as an experimental tool for identifying short-range In clustering. This could be useful for controlling the influence of the growth conditions on the material properties. The significant bowings of *E_g_* in the clustered cases are due to the shortening of the In-N bonds in In-containing alloys compared to the In-N bonds in InN. In the clustered In_0.25_Al_0.75_N alloy, the In-N bonds are approximately equal to 2.02 Å, while in the clustered In_0.25_Ga_0.75_N alloy, they are approximately equal to 2.07 Å (compared to InN, which is approximately 2.15 Å) (refer to Figures 10 and 11 in Ref. [7]). Shortening the bonds results in a stronger interaction at the top of the VB between the states originating from In and the states of the nearest N atoms. This pushes the top of the VB up and significantly decreases the band gap.

Comparing the *dE_g_*/*dp* bowings shown in Figure 8 with the *E_g_* bowings in In*_x_*Ga_1−*x*_N and In*_x_*Al_1−*x*_N shown in Figures 2 and 3 in Ref. [7], it is clear that the *dE_g_*/*dp* bowings are much more significant than the bowings of *E_g_* itself. In general, under hydrostatic pressure, the band gaps increase due to the upward shift of the CB. However, in the clustered case, the shortening of the bonds under pressure further increases the interactions between In and the neighboring N states, leading to an enhanced bandgap reduction compared to the situation at ambient pressure. This means that the resulting pressure coefficients are much smaller than those in the absence of clustering effects. The minimum value of *dE_g_*/*dp* in the clustered arrangement in both alloys should be close to *x* = 0.25 because when a nitrogen atom is surrounded by four indium atoms, the interactions between In and neighboring N states are the strongest [7]. The more pronounced bowing effects in InAlN compared to InGaN can be explained by the shorter bonds in InAlN than in InGaN.

## 3. Pressure Studies of Native Defects and Impurities

The electronic quality of a semiconductor and its importance for device applications are largely determined by the number and character of impurities and native defects. For the realization of high-performance electronic and optoelectronic devices based on nitride semiconductors, it is crucial to understand the role of dopants. Identification of the source of *n* and *p* conductivity is critical. To determine the source of the electron or hole conductivity, it is necessary to investigate all possible defects and dopants. The high-pressure technique is an efficient experimental tool for characterizing native defects and impurities in semiconductors. By comparing the experimental and theoretical pressure coefficients, the hydrostatic pressure can help identify the origin of the different PL spectral lines.

### 3.1. Point Defects in Nitrides—Pressure Effects

With the growing interest in nitrides in the early 1990s, much attention has been paid to identifying the dominant point defects in these materials. The experimental studies [57] were accompanied by ab initio calculations of dopants and native point defects, such as C, Si, Ge, Be, Zn, Mg, O, and H [58,59]; see also review—Ref. [60].

A detailed theoretical study of native defects and some common dopants, including their high-pressure behavior, was performed by Gorczyca et al. [61]. They showed that the bandgap pressure coefficient and the pressure coefficients of the same defect states are similar in AlN and GaN. It was then shown that there is a simple relationship between the position of the defect state in the energy gap and its pressure coefficient. Namely, all the considered defect states can be divided into three groups:

1. A very small pressure coefficient, from −5 meV/GPa to 4 meV/GPa. These are the neutral states of V_cat_, C_N_, Mg_cat_, Zn_cat,_ and *s*-like of V_N_, which are degenerate to VBM or are up to 0.5 eV above VBM, and their charged states, which are up to 2 eV above VBM. As expected, under hydrostatic pressure, the states near the edges of the band follow them.
2. The pressure coefficients are between 9 and 23 meV/GPa. These are all antisites that produce states near the center of the gap.
3. The pressure coefficients are between 12 and 37 meV/GPa. These are the states of V_N_ and C_cat_ that degenerate to or just below the CBM.

Following Ref. [61], the search for dominant defects and impurities in nitrides, especially the search for the dominant donor, has been the subject of many papers.

### 3.2. n-Type Doping—DX Centers in GaN and AlGaN

Donors in III–V compound semiconductors are interesting because of their often metastable nature, which allows them to generate both extended and localized states. A transition from a shallow hydrogenic state to a highly localized state can be induced by hydrostatic pressure or alloying with another group III metal. These effects are associated with the so-called *DX* centers [62]. *DX* centers have been extensively studied since the early 90’s. It is important to understand the electrical and optical properties of *DX* centers under hydrostatic pressure. It was found that the properties of the *DX* level in a metastable state, i.e., when it is resonant with the conduction band, are similar to the properties of the stable substitutional donor. This state was also found to be strongly lattice coupled, with a large energy difference between the thermal and optical ionization energies. Due to the large energy relaxation, the donor can be moved from the substitutional site to the interstitial site with the local variation of the environment. Calculations of the total energy have shown that this configuration is stable when the donor is trapping two electrons (negative U) [62].

In early reports on the dominant donor in GaN, researchers focused on native defects: vacancies and antistites. It first appeared [8,63] that a nitrogen-vacancy might be a good candidate for a residual donor. It is a shallow donor at ambient pressure and introduces a resonance that crosses the bottom of the CB at *p* > 20 GPa. Due to the appearance of a conduction band resonance in the forbidden gap, a freeze-out of electrons from the conduction band should occur.

After further identification of unintentional impurities in nitrides, it has been suggested that these impurities are Si and O donors that can be incorporated into GaN and AlGaN in high concentrations, resulting in unintentional n-type doping. Further analysis revealed that oxygen, not silicon, is responsible for the n-type conductivity in high-pressure-grown GaN [9,64]. Under hydrostatic pressure, the O donor dopant displays the characteristic features of a *DX* defect, while Si behaves as a hydrogenic donor. Oxygen has low energy of formation and acts as a shallow donor occupying a substitutional N site. It is important to note that *DX* center formation has only been observed in the wurtzite structure.

**Figure 9:**
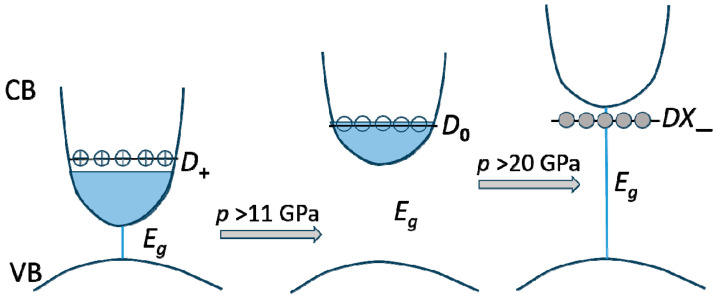
Schematic illustration of the emergence of the resonant *DX* state of oxygen (high doping level) in the gap under pressure.

Under pressure and in the case of a high doping level in the investigated samples, we observe a transition from *D*_+_ through *D*_0_ to *DX*_−_, being positive, neutral, and negative charge states of the oxygen *DX* donor [64]. This process is illustrated schematically in Figure 9. At low pressures (below 11 GPa), the oxygen-related *D*_+_ donor state is formed, which is the additional source of the high-electron concentration. With increasing pressure around 11 GPa, the crossing of this level with the Fermi level and the transition to the neutral *D*_0_ state is observed. With a further pressure increase above the critical pressure of about 20 GPa, the *D*_0_ state emerges into the gap as a deep negatively charged localized level, *DX*_−_, which represents a metastable state with a large lattice relaxation [9,64]. The formation of *DX_−_* is associated with a large rearrangement of the oxygen-donor environment.

The initial position (at ambient pressure) of the energy level of the oxygen *DX* center in GaN has been estimated from the value of the transition pressure from the resonant state to the band gap, and the comparison of the *dE_g_*/*dp* and the estimated pressure coefficient of the localized defect. It has been predicted that the neutral O level is at an ambient pressure of about 0.40 eV above the CBM [9,64]. In the case of dilute doping, electrons autoionize to the CBM and bind to the dopant atom at low temperatures in quasi-hydrogenic states. It is important to note that this process occurs only at low temperatures.

The metastability of oxygen *DX* donors can be illustrated by studying AlGaN alloys. While in GaN, the oxygen-donor forms a classical *DX* center under pressure, in AlGaN, such an oxygen DX center is formed with increasing Al concentration (i.e., with increasing bandgap). The additional application of hydrostatic pressure can also be useful in studying metastability effects.

Doping issues in AlGaN alloys, particularly in the n-type, have significant implications for the production of wide-bandgap devices such as LD, LEDs, UV detectors, and transistors. McCluskey et al. [65] presented theoretical and experimental evidence for the metastable *DX* centers of oxygen donors in unintentionally doped AlGaN. In the samples investigated, the oxygen and silicon concentrations were about 10^19^ and 10^18^ cm^−3^, respectively. An increase in the electron activation energy with increasing aluminum content was observed from the Hall effect measurements. This was consistent with the observation of the *DX* level intersecting the CB at *x* = 0.27, a value estimated from the Hall effect, persistent photoconductivity, and optical threshold measurements.

**Figure 10:**
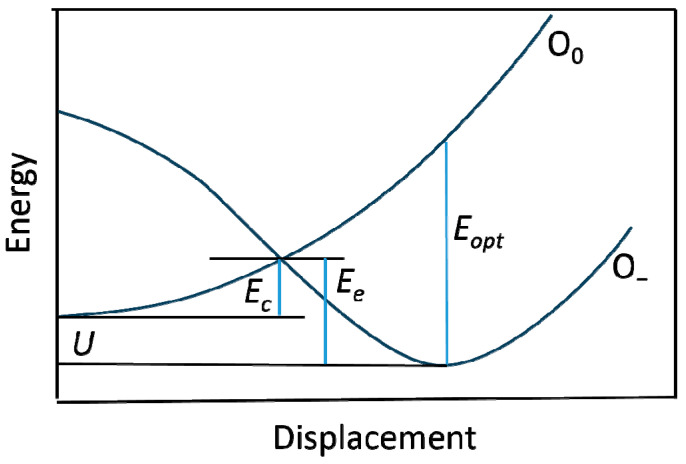
Schematic configuration coordinate diagram for oxygen displacement in AlGaN. The diagram includes the optical ionization energy (*E_opt_*) as well as the capture (*E_c_*) and emission (*E_e_*) barriers. Based on the calculations in Ref. [65].

A schematic coordinate diagram for the displacements of oxygen along [0001] in AlGaN, obtained from ab initio calculations for oxygen in GaN and AlN, is shown in Figure 10. It illustrates the metastable nature of the oxygen DX center. An interpolation was made assuming that the energy of the *DX* configuration is ~0.1 eV lower than that of the substitutional donor (*U*~0.1 eV). The capture and emission barriers were calculated to be 0.4 and 0.5 eV, respectively, in agreement with the experimental values. The ionization energy was found to be 1.3 eV. The details of these calculations are provided in Ref. [66].

To demonstrate the metastable nature of the oxygen *DX* state, persistent photoconductivity can be used [64], or alternatively, high-pressure freeze-out of electrons, as shown in the work of Skierbiszewski et al. [67].

### 3.3. n-Type Doping—Resonant Localized Donor State in InN

At the beginning of the 21st century, growing interest was devoted to the properties of InN. The first epitaxial layers available were highly *n*-type material. Investigations have focused on the existence of a local donor state. Its energy was estimated by studying the Hall electron concentration and Hall mobility [10,11] to be ~80–90 meV above the CBM at ambient pressure. Its pressure coefficient was found to be ~−25 ± 1.0 meV/kbar. This resonant donor state (RDS) determines the electrical properties of the InN samples. Transport studies of n-InN were performed to verify the nature and origin of the RDS [11]. To verify whether these states were formed by native point defects, n-InN samples were irradiated to introduce native donor defects. An increase in the Fermi level position was observed, reflecting an increase in the electron concentration. Hydrostatic pressure was used to verify the number of RDS in the sample during the irradiation runs. These results led to the conclusion that we cannot associate RDS with native point defects. To our knowledge, no further studies have been performed to determine the character of the RDS (whether it is a *DX* state with large lattice relaxation or not).

### 3.4. p-Type Doping—Magnesium Acceptors

Magnesium is the only acceptor that is widely used in the nitride optoelectronics and electronic industries. It is used for *p*-type doping in GaN and related alloys, such as AlGaN and InGaN.

In GaN layers with low Mg doping, the 3.27 eV UV band is observed in optical measurements [68]. This band is commonly attributed to donor-acceptor pair (DAP) recombination, where both the donor and acceptor are shallow impurities. In the case of high Mg doping, we observe the blue light emission at about 3 eV. In order to study the different characteristics of these two bands, high-pressure measurements have been used [67].

The linear pressure coefficient corresponding to DAP recombination at 3.27 eV was found to have a value of 35 meV/GPa, which is close to the GaN bandgap pressure coefficient (~40 meV/GPa).

With a further increase in the Mg concentration (above ~5 × 10^19^ at./cm^3^), the samples became highly resistive. Self-compensation could explain the disappearance of the *p*-type nature of the GaN samples. The possible mechanism of self-compensation could be the formation of up to three deep localized donor states with levels ~0.2–0.8 eV below the CB [68].

To further investigate the different natures of the above mechanisms, hydrostatic pressure is a useful tool for distinguishing between delocalized shallow electronic states and their localized counterparts.

Assuming a weak dependence on m* and the dielectric constant, shallow donor levels follow the CBM as the band gap increases with increasing pressure. This behavior reflects the fact that the shallow donor wavefunction is constructed from the CB minimum wavefunctions. Thus, the pressure coefficient of the shallow donor level should be similar to that of the GaN bandgap (~40 meV/GPa). On the other hand, the pressure coefficients of strongly localized donors should follow a weighted average of the different CB extrema.

In Ref. [68], the pressure evolution of the 3.27 eV DAP band in GaN is 36 ± 0.3 meV/GPa, which is characteristic of shallow-donor-shallow-acceptor pair recombination. The pressure coefficient of the blue luminescence at 3.07 eV was found to be 25 ± 0.3 meV/GPa for bulk GaN crystal samples, which is significantly smaller than that found for DAP recombination. This suggests that the blue band corresponds to a transition from a deep localized donor state to a shallow acceptor state (probably Mg-related).

A disadvantage of Mg doping is the relatively high ionization energy of the Mg acceptor, which makes doping less effective. Hall measurements have shown that the Mg acceptor level in GaN is ~160 meV above the VBM. It is even more difficult to achieve *p*-type material in AlGaN—the ionization energy of the Mg in AlN was found to be quite high ~0.51 eV.

Therefore, researchers have started to look for an alternative acceptor. At the beginning of this century, beryllium was thought to be a promising dopant for obtaining an efficient p-type material. The pressure behavior of Be as compared to that of Mg impurities in GaN, has been studied experimentally and theoretically [69]. The results obtained provided evidence for their identification. It was also feasible to define the similarities and differences between these two types of acceptors. It was clearly shown that they have different properties. Two luminescence bands were found: at 3.38 eV and at about 2.2 eV, which is close to the yellow luminescence in undoped GaN. Also, the pressure coefficient of the Be level (0.8 meV/GPa) is much smaller than that of the magnesium level (~4.8 meV/GPa) [69].

Because of its lower ionization energy, Be is potentially a better *p*-type dopant than Mg. However, to date, there has been no evidence of *p*-type conduction in GaN doped with Be. It remains to be seen why Be, despite having a low binding energy, fails to act as an efficient acceptor in GaN. Too high a concentration of interstitial beryllium, which acts as a compensating donor, may be the main obstacle.

An example of magnesium and beryllium impurities in GaN has shown how the high-pressure technique is useful for identifying impurity states and studying their behavior. By comparing the theoretical and experimental pressure coefficients, the high-pressure technique has been shown to be very helpful in identifying defect states.

## 4. High Pressure in Quantum Wells and Superlattices

### 4.1. Quantum Structures

Quantum heterostructures are layered structures consisting of quantum wells (QWs) and quantum barriers (QBs). They are denoted as QW/QB, where the QW has a smaller *E_g_* value than the QB. Structures with thick QBs are called single quantum wells (SQWs) or multi-QWs (MQWs). A slightly different type of structures are superlattices (SLs), multilayer structures with thin QWs and QBs (usually a few atomic layers). They are also called short-period SLs and are sometimes denoted as *m*QW/*n*QB, where *m* and *n* are the numbers of atomic monolayers (MLs) in the QW and in the QB, respectively. The characteristic feature of the superlattice is the communication between neighboring QWs, which is realized by wavefunctions tunneling through adjacent narrow QBs.

Although the results of the measurements on MQWs are similar to those performed on SLs, their properties are calculated using different methods. SQWs and MQWs are calculated using simulation methods based on the effective mass theory. The band structures of *m*QW/*n*QB SLs, on the other hand, are usually calculated using ab initio methods and the supercell geometry. In these calculations, a supercell containing *m* atomic layers of QW and *n* atomic layers of QB is repeated “ad infinitum”.

All of the above structures represent two-dimensional (2D) objects. However, the high interest of the nitride community, including industry, is also focused on lower dimensional structures such as quantum wires (1D) and quantum dots (0D). In this review, we will concentrate on 2D structures since almost all results on the application of hydrostatic pressure to study the properties of nitride heterostructures are related to this class of objects.

High-pressure studies, which often combine experiments and theory, can provide important results that are otherwise difficult or impossible to obtain and allow for the description of the main factors that influence radiative recombination processes and radiative efficiency. Recent studies [70] have shown that high-pressure spectroscopy is an effective tool for analyzing factors related to strain effects, built-in electric fields, and the involvement of defect states in the recombination processes in quantum heterostructures.

Now, we will focus on the internal electric field (approaching a few MV/cm) present in polar wurtzite structures of nitride heterostructures. In addition to the interesting physics associated with this internal field, it strongly influences the efficiency of light emission in nitride-based devices. In particular, they have been studied under pressure in light-emitting devices.

The built-in electric field originates from two types of polarization, spontaneous and piezoelectric, present in nitride heterostructures with wurtzite symmetry. The **spontaneous polarization** is related to the asymmetric atomic arrangement in the hexagonal unit cell and the resulting charge separation along the *c*-axis of the hexagonal structure.

The **piezoelectric polarization** is due to the strain caused by the difference between the lattice parameters of the QWs and QBs (i.e., the lattice mismatch). It induces a corresponding charge separation along the *c*-axis of the wurtzite structure.

Comparing InGaN/GaN and GaN/AlGaN systems, the difference in ionic radius between Al and Ga is much larger than between In and Ga, leading to a larger charge separation between Al and N atoms and making GaN/AlGaN more spontaneously polarized than InGaN/GaN, where the smaller charge separation leads to lower spontaneous polarization. On the other hand, in InGaN/GaN, the piezoelectric field is high, especially in high In content structures, because of the significant lattice mismatch between the lattice constants of InN and GaN (≈11%). In AlGaN/GaN systems, the lattice mismatch is much smaller (≈3.6%), and the piezoelectric polarization is much lower.

The high spontaneous polarization of GaN/AlGaN makes it suitable for high-temperature piezoelectronics and pyroelectric sensors. Ultraviolet light-emitting devices based on GaN/AlGaN are also very attractive. On the other hand, InGaN/GaN, which has a lower spontaneous polarization and a higher piezoelectric polarization, is widely used in optoelectronic devices such as LEDs, LDs, and solar cells operating in the lower energy range.

**Figure 11:**
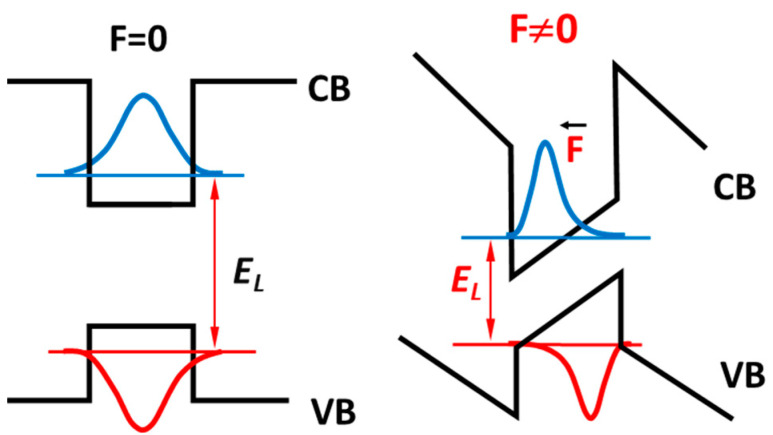
Comparison of the band structure profiles along the *c*-axis of the wurtzite structure for a single QW without (**left**) and with (**right**) an internal electric field. *F* denotes the internal electric field. *E_L_* denotes the luminescence energy.

In QWs, polarization leads to interface charges that induce strong electric fields of up to several MV/cm along the polar *c*-direction. They strongly affect the quantum structure properties and performance of the devices based on them. As an effect of the built-in electric field, we observe the tilting of the heterostructure band profiles and the associated shift of the wave functions to the interfaces of the QW, which reduces the wave function overlap. The above effects are shown schematically in Figure 11. They lead to (i) a reduction in the emission energy (“red shift”), equivalent to an increase in the emitted light wavelength, and (ii) a reduction in the emitted light intensity (reducing wavefunction overlap reduces radiative recombination). The above phenomena caused by the internal electric field (*F*) are called the Quantum Confined Stark Effect (QCSE). It describes the increase in potential in the QW by the equation Δ*V = F*·*L*, where *L* is the width of QW.

Hydrostatic pressure increases the internal electric field strength [71]. The piezoelectric effects can be studied by means of high-pressure spectroscopy on the basis of the following relationship [70]:

(2) $$E_{PL}=E_{g}+E_{e}+E_{h}-q\cdot L\cdot F-E_{x}$$

where *E_PL_* is the transition energy between the confined states in the QW. *E_g_* is the energy gap, *E_x_* is the exciton binding energy, *E_e_* and *E_h_* are the confinement energies of the electron (*e*) and hole (*h*) levels, *q* is the electric charge, *L* is the QW width, and *F* is the internal electric field. The pressure derivative of Equation (2), assuming a weak pressure dependence of confinement and exciton energies, can be approximated by

(3) $${dE}_{PL}/dp={dE}_{g}/dp-q\cdot L\cdot dF/dp$$

Thus, by measuring the pressure coefficient of the PL energy, *dE_PL_*/*dp*, we can obtain the pressure dependence on the built-in electric field and on the QW width. However, these two factors are not independent, but it is possible to separate them by playing with the QW width or the QW/QB width ratio.

Perlin [72,73], Shan [74], and Vaschenko [75,76] reported a significant decrease in *dE_PL_/dp* in nitride QWs. According to Łepkowski et al. [77,78], this phenomenon should be studied in terms of the pressure dependence of the piezoelectric field, including nonlinearity effects. Their study found a significant strain dependence of the piezoelectric coefficients. An investigation of the *E_PL_* and *dE_PL_*/*dp* in InGaN/GaN QWs as a function of laser power was recently published by Staszczak et al. [79]. The performed experiment revealed the process of almost complete screening of the internal electric field in the studied quantum well. The emission energy and its pressure coefficient exhibited saturation above a certain laser power density. The *dE_PL_*/*dp* reached the value characteristic of the InGaN alloy layers used for the construction of the studied QW.

Despite recent intensive research on this topic, knowledge of the polarization effect and internal electric fields is not sufficient, and there are still results that have not been confirmed by experiments [80,81,82,83,84,85,86,87]. This is in part because experimental studies often involve QW structures containing InGaN or AlGaN alloys, where inhomogeneity, segregation, and chemical ordering are present. To overcome this difficulty, samples of binary nitride multi-quantum wells (MQWs) have been synthesized and experimentally studied [88]. The correlated theory and experimental data from the study of these samples provide insights into the properties of polar nitride MQWs and provide a basis for predicting the optical properties of other polar quantum structures and devices. In addition to the strain and electric field, lattice mismatch leads to the generation of structural defects and can alter the incorporation of impurities, thereby affecting the emission of QWs [88]. As mentioned before, hydrostatic pressure spectroscopy allows the distinction between band-to-band radiative transitions and those involving deep or shallow defect states [88,89,90,91,92,93]. Transitions from a shallow donor to the VB state or band-to-band transitions occur when the pressure dependence of the PL is similar to that of the band gap. When considering deep donor-acceptor or donor-VB state transitions and inter-defect state transitions, a much weaker pressure shift of the *E_PL_* is observed because the deep states are constructed from the wave functions of the whole BZ and are characterized by a weaker dependence on pressure than the band-to-band transitions.

Spectroscopic studies of the influence of pressure on the luminescence properties of nitride QWs confirmed a dramatic change in the PL dependence on the applied pressure, QW geometry, crystallographic orientation, and lattice mismatch between QWs and QBs or QWs and substrates [88,94,95,96,97]. The collected data were compared with calculations of the electronic properties of the studied structures. The main results and conclusions from the pressure studies of wurtzite QW structures GaN/AlN, GaN/AlGaN, and GaN/AlInN with different QW thicknesses and various QB compositions grown along the polar and nonpolar wurtzite directions are summarized below.

### 4.2. GaN/AlN QWs

GaN/AlN QWs are the only binary wurtzite QW systems that have been studied experimentally. The theoretical study of InN/GaN SLs under pressure has been published by Gorczyca et al. [97], but as will be shown later, growing a pure InN/GaN quantum structure is not yet possible.

Measurements of the optical properties of GaN/AlN QWs at ambient and high hydrostatic pressures were compared with ab initio calculations performed on analogous structures [88]. Analysis of the obtained results included the dependence of the PL on the QW thickness, the influence of strain, electron screening, and defect states.

In agreement with Equation (2), a red shift of the PL spectra to energies below the band gap of GaN, caused by the electric field parallel to the growth direction, was observed with increasing QW width according to the QCSE. As a consequence, the recombination rates also decrease for wider QWs, even by several orders of magnitude. Theoretical calculations of the electronic band structure included MQWs strained to AlN, GaN, or the experimentally determined lattice constant. Two cases were studied: no doping and n-doping with an experimental charge concentration [88]. For details of the theoretical calculation, see also Refs. [93,98,99].

Theoretical results for narrow QWs match experiments. However, for wider QWs, there are discrepancies between the calculated low oscillator strengths and higher experimental decay rate [70]. The experimentally observed decay, which is too high compared to the calculations, could be caused by the screening of the internal electric field by free carriers. This effect increases the overlap of the electron-hole wave function due to the reduction of the electron-hole distance, thus increasing the experimentally observed decay rate. The observed decay could also be due to the influence of PL from regions where the QW width was reduced by thickness variations. Another explanation is the influence of defect states, which are more pronounced in wider QWs.

We can distinguish between the above factors using the high-pressure technique. Comparing the PL peak pressure coefficient versus GaN/AlN QW thickness with theoretical results shows the following:

(i) For thin GaN/AlN QWs (1–4 nm), the experimentally determined *dE_PL_*/*dp* values are in good agreement with theoretical predictions [88,98]. They decrease from +24 meV/GPa (1 nm QWs) to −22 meV/GPa (4 nm QWs) because of the increase in the internal electric field with pressure according to Equation (2). This confirms the correctness of the theoretical model in the description of the electronic properties of the polar QWs.
(ii) For thick GaN/AlN QWs (6 nm), the measured *dE_PL_*/*dp* = −3 meV/GPa differs significantly from the theoretical value of ~−60 meV/GPa. This is likely an indication of emission from a deep defect state, which is more efficient than QW PL.

Also, the decrease in PL energy and decay rate with increasing QW width has been quite accurately reproduced by ab initio calculations. The calculations [70] showed that the strain-induced effects associated with the lattice mismatch between the substrates and the MQW systems are responsible for the observed dramatic reduction in the *dE_PL_*/*dp* in GaN/AlN MQWs. These effects allowed to describe the increase in the built-in electric field with hydrostatic pressure in these systems [99]. For details, see Ref. [70].

From the above analysis, it can be seen that the high-pressure technique is a valuable research tool for identifying optical transitions.

### 4.3. GaN/AlGaN QWs

Studying the ternary QW structures, such as GaN/AlInN and GaN/AlGaN, is more complicated. Different atomic distributions with a tendency toward clustering or segregation can substantially influence the properties of AlGaN and InGaN alloys, and they should be taken into account. A high-pressure study of a series of GaN/Al*_x_*Ga_1−*x*_N QWs samples with layer thicknesses of QWs and QBs of about 3 mm and 4 mm, respectively, was reported in Refs. [70,96].

It was found that the dependence on the Al content, *x*, was particularly strong under pressure. The pressure coefficients of the emission energies *dE_PL_*/*dp* decrease significantly with increasing *x* values, from ~35 meV/GPa for *x* = 0.25 to ~−8 meV/GPa for GaN/AlN.

PL emission energy depends on the doping level. A good agreement between the experiment and theoretical ab initio calculations was found for a doping level of *n*~7 × 10^18^ cm^−3^.

In contrast to the PL emission energies, the dependence of *dE_PL_*/*dp* on the Al content in the QB is less sensitive to the doping level. Both theory and experiments confirmed that the main factor responsible for the strong reduction in *dE_PL_*/*dp* is caused by nonlinear effects resulting from the internal strain due to the substrate and the QW lattice mismatch.

In addition, layer thickness variations, alloy fluctuations, blurred QW-QB interfaces, and shallow defect states cannot be neglected [89].

It has also been shown in Ref. [70] that with increasing Ga content in the QB, the electric field in the investigated structures decreases, which increases the emission efficiency. At the same time, the QB height and the carrier quantum confinement in the QW are reduced.

**Figure 12:**
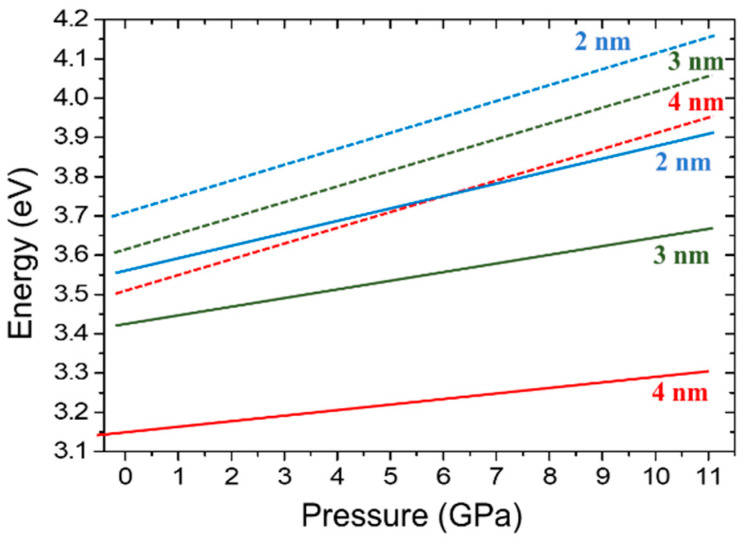
(Color online). Schematic dependence of the PL peak position on pressure in GaN/Al_0.3_Ga_0.7_N for different QW thicknesses. Two cases are illustrated: QWs grown along the nonpolar (dashed lines) and polar (solid lines) crystallographic directions. Based on Figure 6 in Ref. [70].

To compare the optical properties of the structures without and with the presence of an internal electric field, GaN/Al_0.3_Ga_0.7_N QWs grown along nonpolar and polar wurtzite directions were reported in Ref. [95]. Both structures of the same geometry contained three GaN QWs of different widths that were separated by thick Al_0.3_Ga_0.7_N QBs. The results of high-pressure measurements of the above polar and nonpolar QWs structures are shown schematically in Figure 12. In the polar samples, the pressure coefficients are much smaller, and a red shift of PL emission energies is observed with increasing QW thickness due to the QCSE. On the other hand, the *dE_PL_*/*dp* coefficients of the nonpolar QWs are nearly the same due to the absence of an internal electric field, so the transition energies are defined by the QW confinement effects [Equation (2)], and the *dE_PL_*/*dp* can be described by Equation (3).

Later (in 2022), the effect of the internal electric field on the pressure behavior of polar and nonpolar GaN/AlGaN MQWs with the same composition was investigated by Koronski et al. [100]. Differences in the pressure behavior of the PL in the nonpolar and polar MQWs were observed. In nonpolar structures, the pressure dependence follows the *dE_g_*/*dp* of GaN. On the other hand, the *dE_PL_*/*dp* in polar MQWs was strongly reduced with respect to the *dE_g_*/*dp* of bulk GaN, which can be explained by the pressure-induced increase in the piezoelectric field and by the nonlinear effects. The above experimental results agree with the theoretical predictions.

As mentioned above, high-pressure experiments demonstrated that the internal electric field in polar structures increases under pressure, causing a pronounced redshift of the PL and a reduction in the quantum efficiency according to the QCSE. The decrease in emission efficiency for wider QWs can lead to defect-derived emissions with a very weak dependence of the transition energy on pressure.

Finally, it has been shown that in polar samples, the changes in *dE_PL_*/*dp* with QW thickness are caused by the pressure-induced increase in the internal electric field.

**Figure 13:**
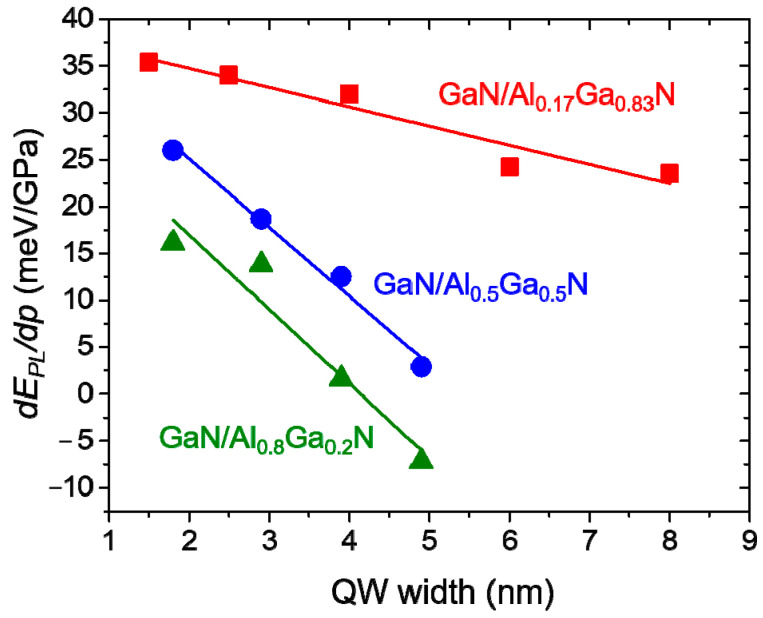
Dependence of the *dE_PL_*/*dp* on the QW width for QWs in GaN/Al*_x_*Ga_1−*x*_N with different Al concentrations, *x* (Ref. [76]).

Another example of GaN/AlGaN QWs studies under pressure comes from Ref. [76]. PL measurements under pressure were performed for different samples with Al concentrations of *x* = 0.17, 0.5, and 0.8, and various QB widths. The resulting dependence of *dE_PL_*/*dp* on the QB width for the three samples is shown in Figure 13. It can be concluded that the increase in both Al content and QW width leads to an increase in the PL pressure coefficient, corresponding to the increase in the built-in electric field.

### 4.4. GaN/AlInN QWs

Improvements in the growth of AlInN layers have allowed for detailed studies of this material. The AlInN bandgap covers a spectral range from the infrared (InN) to the UV (AlN), which is of great importance for photonic and electronic applications. GaN/AlInN MQW structures can be an alternative to GaN/AlGaN systems for the production of UV emitters.

Low resistance and good mobility properties enable the fabrication of devices such as high-electron mobility and field-effect transistors. The large CB offset between AlInN and GaN allows the fabrication of devices operating in the spectral range from 2.5 µm to 4 µm.

**Figure 14:**
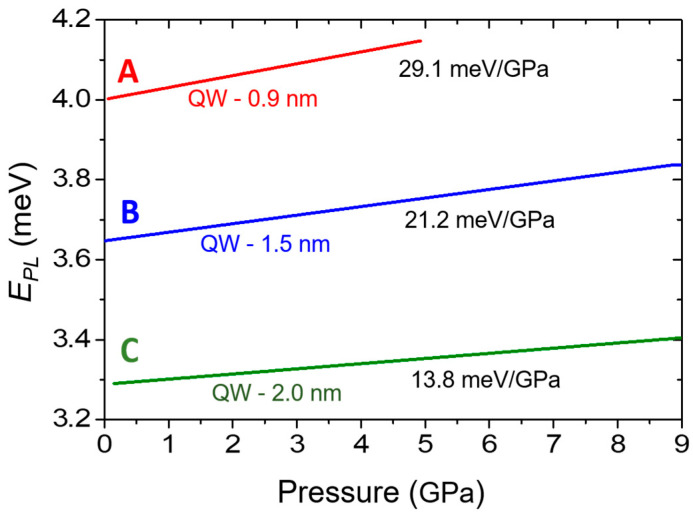
Pressure dependencies of the *E_PL_* for three samples (A–C) of GaN/Al_0.88_In_0.12_N QWs. The values of the QW widths and pressure coefficients are given in the plots. Based on Figure 8 in Ref. [70].

An important advantage of the AlInN alloy is that 17% of indium is lattice-matched to GaN. Lattice-matched GaN/AlInN structures can eliminate piezoelectric polarization [100,101,102,103]. However, the effect of spontaneous polarization must still be considered. To analyze the pressure-induced piezoelectric effects in lattice-matched GaN/AlInN QWs, PL measurements were performed on three samples of GaN/Al_0.88_In_0.12_N MQWs with various QW widths and a constant QW/QB ratio of ~0.45 [94]. In Figure 14, the pressure dependencies of the PL peak positions of these samples, labeled A, B, and C, are shown schematically by lines fitted to the results of the measurements of the energy peak positions under pressure. Similar to GaN/AlN MQW systems, a red shift of PL energies with increasing QW thickness was observed according to QCSE [14]. Two separate regions with different values of *dE_PL_*/*dp* can be identified. Up to 9 GPa, the PL peak energies increased with increasing pressure for all three samples. The pressure coefficients, which are smaller for wider QWs, are ~29.1 meV/GPa, ~21.2 meV/GPa, and ~13.8 meV/GPa for samples A, B, and C, respectively. This behavior, similar to that in GaN/AlN MQWs, indicates the presence of an electric field that increases with pressure due to changes in spontaneous polarization.

The values of the internal electric field in GaN/AlInN QWs and its pressure dependence were determined by comparing the measured dependence of the *E_PL_* as a function of the QW thickness with the dependence obtained theoretically based on Equation (2) and solving Schrödinger’s equation for a triangular QW [94]. The obtained values of the internal electric field in GaN/Al_0.88_In_0.12_N were equal to ~4 MV cm^−1,^ and its pressure coefficient was ~0.29 MV/(cm GPa) with a qualitative agreement with the theoretical value of ~0.17 MV/(cm GPa) [94].

In conclusion, the pressure behavior of the *E_PL_* vs. QW width of GaN/Al_0.88_In_0.12_N QWs is quite similar to that of GaN/AlGaN QWs [75,104,105,106]. This means that despite the minimization of the piezoelectric component at ambient pressure, there is still a pressure dependence of the piezoelectric effects and a large internal electric field resulting from the spontaneous polarization. Thus, the pressure properties of lattice-matched QW systems are not fundamentally different from those of lattice-mismatched systems.

### 4.5. InGaN/GaN QWs

InGaN/GaN QWs and SLs are the building blocks of LEDs and LDs operating in the blue, green, and UVA spectral regions. In general, the emission wavelength of the device can be modulated by changing the In content in the QW; for blue emission, it should be ~18% In, and for green emission, it should be ~25% In. However, in reality, it is very difficult to grow high In content InGaN layers of sufficient quality, mainly because of the GaN-InN lattice mismatch and the phase separation for In*_x_*Ga_1−*x*_N with *x* > 0.25. These problems are partly responsible for the “green gap” phenomenon.

As was already shown in this chapter, the built-in electric field present in wurtzite quantum structures is responsible for a pronounced shift in the light emission energy and a decrease in the light efficiency, which are the effects described by QCSE. An example of InGaN/GaN QWs will be used in this section to present the high-pressure method for determining the presence or absence of the built-in field.

**Figure 15:**
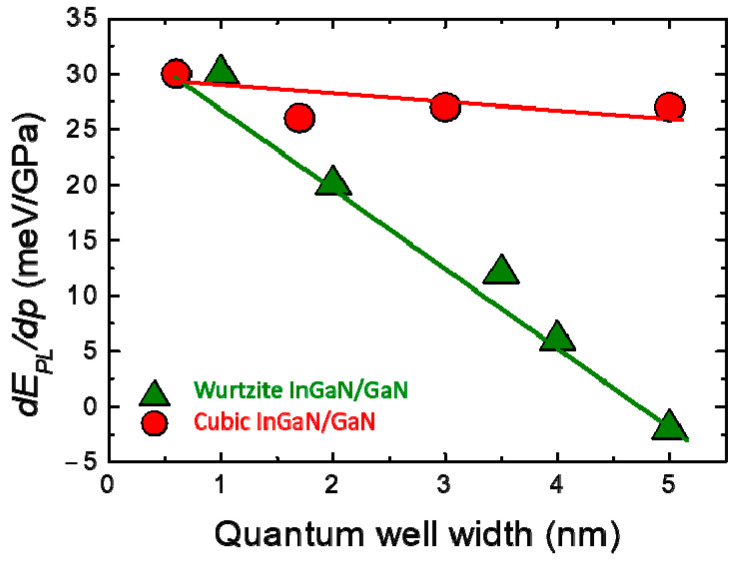
PL pressure coefficients of wurtzite (Ref. [75]) and cubic (Ref. [107]) In_0.20_Ga_0.80_N/GaN QWs as a function of the QW width.

In analogy to Figure 12, where polar and nonpolar GaN/AlGaN structures were compared, Figure 15 shows the results of the high-pressure PL measurements performed on the In_0.2_Ga_0.8_N/GaN QW samples grown in the wurtzite structure (Ref. [75]) and in the cubic structure (Ref. [106]). A strong decrease in the pressure coefficient in wurtzite QW structures with increasing QW width is observed. This effect corresponds to the increase in QCSE, which is proportional to the QW thickness and is almost completely reduced for very thin QWs. In fact, for the case of wurtzite InGaN/GaN with a QW equal to 1 nm, the *dE_PL_/dp* is the same as that for the cubic heterostructure, characterized by the absence of the built-in electric field.

**Figure 16:**
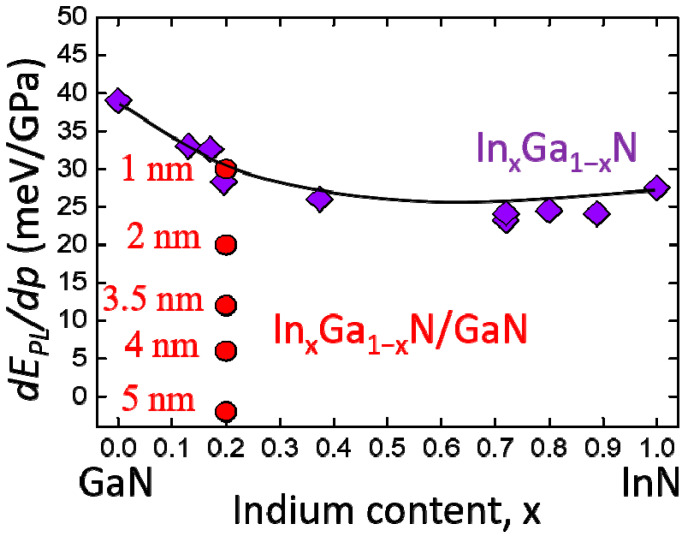
Comparison of the *dE_PL_*/*dp* for InGaN alloy and for InGaN/GaN QWs for different QW widths (as described in the Figure). Experimental data are from Ref. [5] (for the InGaN alloy) and Ref. [75] (for InGaN/GaN QWs). The polynomial fit to the theoretical *dE_g_*/*dp* values for the InGaN alloy is shown by the solid line.

Another example of using hydrostatic pressure to “monitor” the effects of the internal electric field (or QCSE) is shown in Figure 16. The PL pressure coefficients of the set of In_0.2_Ga_0.8_N/GaN QW samples with various QW thicknesses, from 1 to 5 nm, are compared with the *dE_PL_*/*dp* dependence measured on the thick layers of In*_x_*Ga_1−*x*_N.

From the above examples, we can see that the value of the PL pressure coefficient of the quantum heterostructure can monitor the strength of the internal electric field present in this structure.

**Figure 17:**
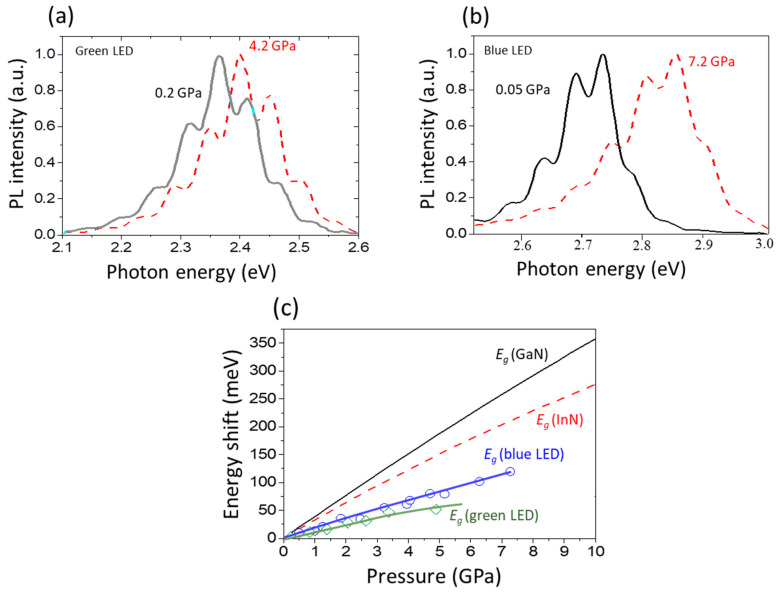
Photoluminescence spectra of (**a**) green SQW LED. (**b**) blue SQW LED measured at different pressures. Spectra are normalized for clarity. (**c**) PL peak positions as a function of pressure for blue (squares) and green (triangles) SQW LEDs compared to the pressure dependence of the InN and GaN band gaps.

The influence of pressure on the PL properties of nitride QWs was investigated by Perlin, Iota, Weinstein et al. in 1997 [72]. The pressure dependence of the PL spectra was measured for the two types of GaN/InGaN/AlGaN LEDs. They found unusually low PL pressure coefficients, *dE_PL_*/*dp*, equal to ~12 meV/GPa and ~16 meV/GPa for the green and blue LEDs, respectively. These values are much smaller than the bandgap pressure coefficients of GaN (~40 meV/GPa) and InN (~25 meV/GPa). The PL spectra for the two selected pressure values are shown in Figure 17a,b. The peak positions of the PL as a function of pressure for both SQW LEDs are shown in Figure 17c. As a possible explanation for the observed effects, the authors suggested that localized states are involved in radiative recombination.

Another explanation for the extremely low *dE_PL_*/*dp* in InGaN-based QWs appeared in 2001 in the paper by Perlin, Gorczyca, Suski et al. [73], where the influence of hydrostatic pressure on the emission and absorption spectra measured for different types of InGaN quantum structures was investigated. The authors found that with increasing In concentration, both *E_PL_* and *dE_PL_*/*dp* values decrease. As a result, *dE_PL_*/*dp* reaches zero for *E_PL_*, which is equal to about 2 eV. As a possible mechanism for this effect, the influence of internal electric fields has been discussed.

The most recent theoretical results on the influence of pressure effects on exciton states in InGaN/GaN coupled double QWs were reported by Wang and Duan [108]. They showed that hydrostatic pressure induces a significant increase in the emission energy and a linear increase in the internal electric field strength.

As an example of another interesting property of InGaN QWs, we can mention the work of Łepkowski and Bardyszewski [109]. They theoretically studied the pressure dependence of the topological edge states in InN/GaN and InGaN/GaN QWs. They showed that by applying hydrostatic pressure, it is possible to drive the system continuously from the insulator state to the semimetallic phase.

### 4.6. In(Ga)N/GaN SLs

The idea of InN/GaN SLs was introduced by Yoshikawa et al. [110]. They pointed out the advantages of SLs over the standard QWs. Superlattices offer the possibility of more effective bandgap engineering by appropriate choice of QW and QB thicknesses, precise control of lattice mismatch strain, and the possibility of reducing the internal electric field by using very thin QWs. Following their work, studies (mainly theoretical) on the electronic and optical properties of InN/GaN SLs [111,112,113,114,115,116,117] began to appear.

The main mechanisms in bandgap engineering were pointed out, which, in In-thin QW SLs, was the hybridization of the QW and QB wavefunctions. In mInN/nGaN SL, this mechanism leads to an increase of the band gap from 0.65 eV (InN) to ~2.1 eV in 1InN/*n*GaN SL. In SLs with thicker QWs, the band gap decreases with increasing QB thickness *n* and reaches zero when the number of QB MLs *m* is greater than five [114]. This effect provides hope for obtaining a topological insulator. The effect of “metallisation” is caused by the existence of internal electric fields, which have an influence on the SL bandgap for wider QWs. The observed red shift in the emission light is in agreement with the QCSE.

Advances in epitaxial growth techniques enabled the first *m*InN/*n*GaN SLs to be synthesized [110,117,118]. They were thin QWs (up to 5 ML). Unfortunately, the PL measurements performed on all the fabricated samples were in significant disagreement with the calculated band gap values. Measured *E_PL_* values were found to be similar to the GaN band gap (~3.4 eV [110,117] and 3.26 eV [118]), while theoretical *E_g_* values ranged from 0 to 2.2 eV [114,115,116,117].

Also, the experimentally obtained *dE_PL_*/*dp* values for the 1InN/*n*GaN SLs samples were much higher than the calculated values. Depending on the number of barrier MLs, the experimental values were in the range of 28.7–33.4 meV/GPa, while the calculated values were found to be in the range of 21–23 meV/GPa.

**Figure 18:**
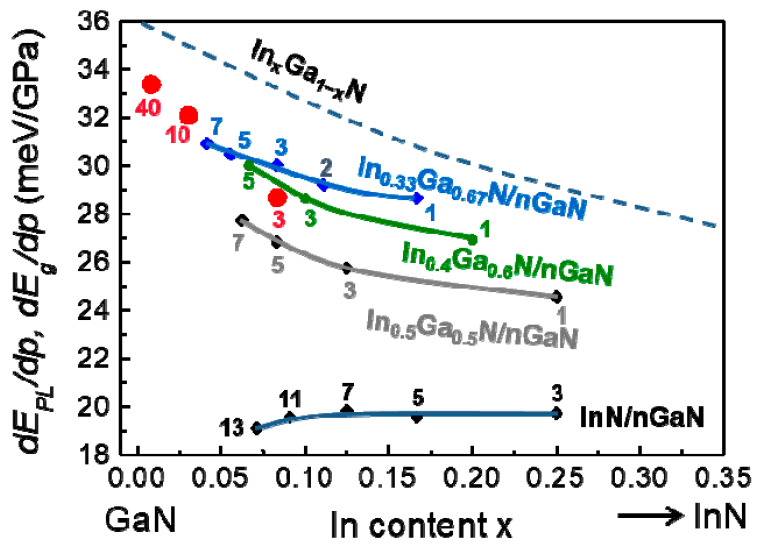
Band gap pressure coefficients of 1In*_x_*Ga_1−*x*_N/*n*GaN SLs versus In content, *x*. Calculated results are indicated by diamonds, and experimental data by circles. The values of *n* are indicated. Lines are spline fits for guidance. Reproduced with permission from Ref. [116] (Figure 27). Copyright 2018 IOP Publishing CC BY licence.

Several hypotheses have been proposed [117,119] to explain these discrepancies. One of them is that GaN excitons are involved in the optical transitions, but the experimentally obtained *dE_PL_*/*dp* was found to be smaller (~30 meV/GPa) than predicted for GaN excitons (approximately 40 meV/GPa). These discrepancies have been the motivation for further investigations of InN/GaN SLs, both theoretically and experimentally. One of the other hypotheses is that Ga atoms can diffuse from the QB region to the QW, and instead of InN QW, we have InGaN QW. To test this hypothesis, the calculated values of the band gaps and their pressure coefficients for different *m*InGaN/*n*GaN SLs were compared with the PL results for nominal *m*InN/*n*GaN SL with *n* = 3, 4, 10, and 40. The results for the pressure coefficients are shown in Figure 18. It can be seen that the best agreement is obtained between the *E_PL_* measured on nominal InN/GaN samples (red dots) and the calculated band gaps of the In_0.33_Ga_0.67_N/GaN SL.

A convincing explanation for the observed discrepancies appeared shortly thereafter in a paper by Suski et al. [15]. The explanation was based on the performed quantification of the In content in the InN/GaN SL in the fabricated [110,117,118] samples used for PL experiments [110,117,118,119]. This procedure was performed using quantitative high-resolution transmission electron microscopy (TEM). It was surprisingly found that the investigated samples consisted of an In*_x_*Ga_1−*x*_N ML with an In content of *x* = 0.33 instead of *x* = 1, as intended. In light of this finding, the very good agreement between the *E_PL_* measured on nominal InN/GaN samples (which turned out to be In_0.33_Ga_0.67_N/GaN samples) and the calculated bandgaps of the In_0.33_Ga_0.67_N/GaN SL shown in Figure 17 is fully understandable.

In conclusion, it was found that the discrepancy between the theory and the experiment was caused by the fact that the real content of indium in the investigated samples of InN/GaN SLs was lower than the intended value. Moreover, it was suggested that it is currently not possible to achieve In*_x_*Ga_1−*x*_N/GaN SL with In content higher than 33%. These findings were confirmed theoretically by Duff et al. [16] and Lymperakis et al. [17], who found that there is an upper limit to the In content in the pseudomorphic growth of InGaN on the GaN substrate. In their calculations, 25% of In was the upper limit for stable In content. The explanation was related to lattice deformation due to lattice mismatch, which involves a high strain energy. At the same time, it has been suggested [16] that it is possible to grow InN on an In_0.25_Ga_0.75_N substrate, which has a higher lattice parameter more similar to that of InN, which reduces the misfit strain.

## 5. Devices and Perspectives

In this section, the role of hydrostatic pressure in the design and characterization of optoelectronic devices will be briefly described, along with some perspectives for future research in the field of this review.

### 5.1. Devices

The nitride-based devices, due to their wurtzite structure, exhibit several properties that are not present in nonpolar semiconductors. This chapter describes some examples of effects where the application of hydrostatic pressure has allowed us to understand and monitor issues related to the presence of an internal electric field in the active part of nitride LEDs and LDs. In optoelectronic devices, the effects of the built-in electric field and QCSE are particularly visible. Electric field engineering to suppress the “harmful” role of QCSE in these devices is realized in different ways by choosing structures with very thin (<1 nm) or alternatively thick (≥10 nm) QW layers in the active area of the device. In the previous chapter, it was shown that for very thin QWs (~1 nm), the effect of the built-in electric field is already negligible. In contrast, this chapter describes the process of QCSE elimination in thick QW layers.

The concept of thick QWs is interesting because they exhibit radiative recombination only from the excited states. In this case, the elimination of the electric field is achieved by effective screening by carriers in the ground states generated by the driving current. The monitoring of this process using a high-pressure approach is the subject of this section. Similar to the study of QCSE in QWs, high pressure was used to provide unequivocal evidence of the presence or absence of QCSE in the material being investigated. The approach used was to compare the hydrostatic pressure coefficient of the electroluminescence (EL) energy, *dE_EL_*/*dp*, with the pressure coefficient of the same material in the absence of an internal electric field. The situation in which they are equal corresponds to a completely screened or non-existent internal electric field. A more detailed explanation of the method used can be found in Refs. [70,120].

**Figure 19:**
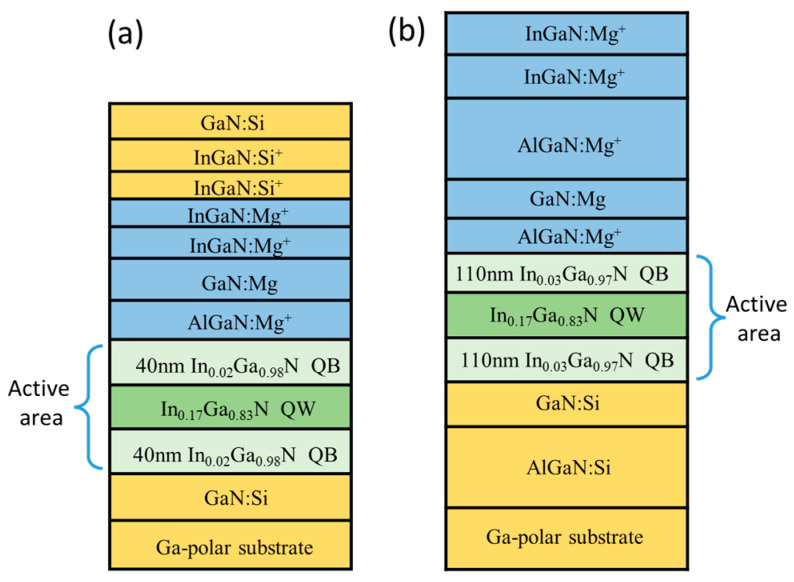
Schematic structures of (**a**) LED with tunnel junction above the QW and (**b**) LD structure with AlGaN cladding to form the waveguide. In both structures, the active layer consists of In_0.17_Ga_0.83_N QW.

The mechanism of internal electric field screening by free carriers is illustrated by the example of some samples of LED and LD with different QW widths [121]. The scheme of their structures proposed by Muziol et al. [122] based on single undoped QWs of In*_x_*Ga*_1−x_*N/GaN with *x* = 0.17 is shown in Figure 19. The choice of the same In concentration in the QW of the proposed LEDs and LDs leads to the introduction of the same piezoelectric field operating in the active region of the studied devices.

The strong dependence of the wavelength and emitted light intensity on certain structural solutions and on the driving current represents the interesting specificity of optoelectronic devices. Now, we will show how the dependence on the driving current and changes in the width of the QW significantly modify the QCSE and, consequently, the properties of the considered emitters and how the hydrostatic pressure can effectively control these effects.

**Figure 20:**
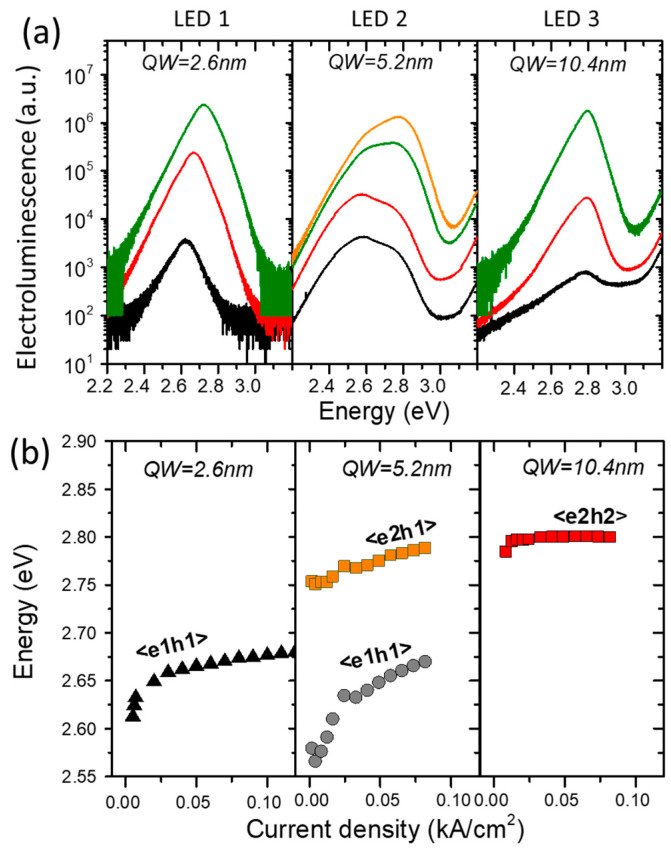
(**a**) EL spectra for LED1, LED2, and LED3 measured at different driving currents, (**b**) EL energy vs. current density for the studied LEDs. Different colors correspond to transitions between different electronic states.

Figure 20 shows the results of EL measurements performed on three types of LEDs with different QW widths: 2.6 nm, 5.2 nm, and 10.4 nm (marked as LED1, LED2, LED3) [123]. All of them have the structure shown in Figure 19 and were grown by MBE on GaN substrates.

Figure 20a illustrates the comparison of electroluminescence spectra (EL intensity vs. energy) measured for some driving currents from 2 to 100 A/cm^2^ in the three studied LEDs. In the case of LED1 and LED2, we can observe a shift of the EL maxima with the driving current to higher energies. However, LED3 does not show any dependence of the EL energy on the driving current. Moreover, the character of the studied dependences shows “one-peak” behavior in the case of LED1 and LED3, while in the case of LED2 “two-peak” character of this dependence is observed.

Identification of the optical transitions between different states of VB and CB was performed using the SiILENCE 5.4 package [5,120] (see Figure 20b). It can be seen that the complete screening of the QCSE by the free carriers generated by the driving current (ID) is obtained for LED3.

**Figure 21:**
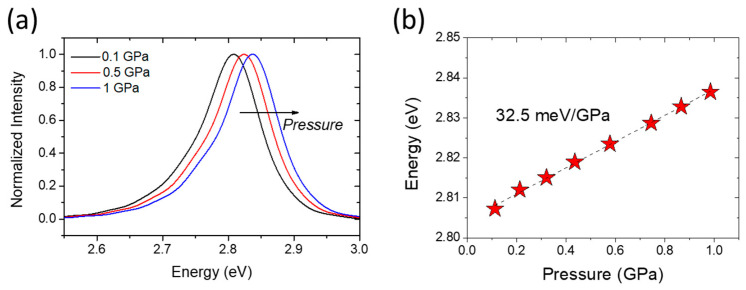
(**a**) Normalized *E_L_* spectra measured in LED3 for three selected values of the applied hydrostatic pressure: 0.1 GPa, 0.5 GPa, and 1 GPa. (**b**) Pressure dependence of the maxima of the *E_L_* spectra for the pressure range of 0.1–1.0 GPa.

To show how hydrostatic pressure can effectively monitor the effects of QCSE suppression by screening, Figure 21a illustrates the behavior of the normalized *E_L_* spectra in LED3. They are shown for three selected values of the applied hydrostatic pressure of 0.1, 0.5, and 1 GPa. A shift of *E_L_* to higher energies is observed with applied pressure. The associated physical effect reflects the pressure-induced increase in the bandgap of In_0.17_Ga_0.83_N.

**Figure 22:**
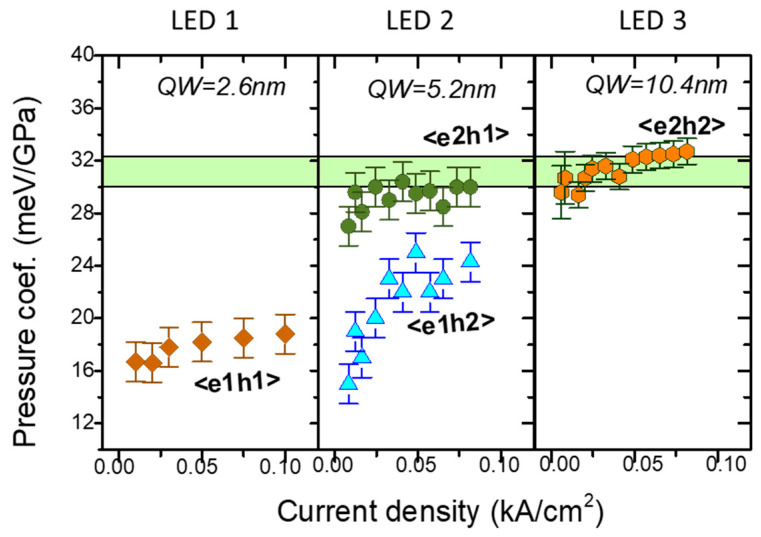
Pressure coefficient of the *E_L_* emission energy vs. driving current density in the studied LEDs. The energetic states of electrons and holes involved in radiative recombination are shown.

Figure 21b shows the pressure dependence (in the range of 0.1 to 1 GPa) of the maxima of the *E_L_* spectra. This effect is equivalent to the band gap shift with pressure. The resulting value of 32.5 meV/GPa is equal to the band gap shift with pressure in the absence of a built-in electric field, which means it is the case of full screening of the internal field. Figure 22 shows the dependence of the evolution of the pressure coefficients obtained from the measurements of the emission spectra as a function of the driving current density at different applied pressures [120]. The light green bar corresponds to the pressure coefficient value that represents a fully screened electric field, *F.*

In the case of LED1, the pressure coefficient increases slowly with the driving current. It can be seen that a large internal electric field determines *E_L_* in the case of this LED with a narrow QW. This shows that the screening of QCSE by carriers supplied by the driving current is not very efficient in this case. For LED2, two optical transitions are observed. The lower one shows the electric field remaining in the active area of LED2, while the upper one corresponds to a partially screened electric field. For LED3 with a QW of 10.4 nm width, only one transition channel is observed. The built-in electric field is completely screened. The obtained results show a very weak dependence of the pressure coefficient on the driving current.

**Figure 23:**
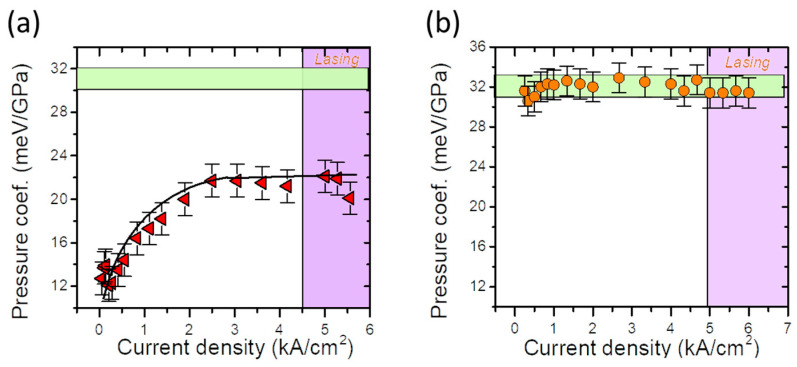
Current density dependence of pressure coefficients for two LDs with QWs of different widths: (**a**) 2.6 nm and (**b**) 10.4 nm. The light green horizontal bars represent the pressure coefficient values corresponding to a fully screened built-in field. The violet bars represent the driving current range above the laser threshold.

Figure 23 shows the dependence of the evolution of the pressure coefficients with increasing driving current density for two LD samples with QW widths of 2.6 nm (Figure 23a) and 10.4 nm (Figure 23b). These dependencies were obtained from measurements of the emission spectra as a function of the driving current density at different applied pressures [120]. From Figure 23, we can see that the behavior of the LDs under pressure is very similar to that of LEDs with analogous QW widths. The same values of the pressure coefficient (~32 meV/GPa) were obtained for LEDs and LDs in the case of total screening of the electric field [120,123].

Further work on the pressure dependence and influence of internal electric fields on the optoelectronic properties of InGaN/GaN-based LEDs has been published in Refs. [124,125,126].

### 5.2. Perspectives

From this review, it can be seen that the physics and technology of nitride semiconductors are developing at a rapid pace and are one of the fastest-growing areas of modern semiconductor research, with the potential to revolutionize various technologies in the coming years. The most advanced areas are represented by visible light devices such as LEDs and LDs. However, electronic devices, such as high-power transistors and vertical transistors, also have great potential, although they were developed and introduced to the industry later than light emitters.

Recently, more advanced measurement techniques have become available, allowing experimental results to be obtained with greater precision. At the same time, more advanced theoretical models based on ab initio methods have been developed. The development of advanced theoretical models and computational tools to understand and predict the behavior of nitride semiconductors at the atomic level is critical for further progress. This will enable researchers to design materials and devices with better-tailored properties. Pressure studies, along with theoretical analysis, can be very useful in understanding and controlling the optical performance of semiconductors.

Future high-pressure studies should focus on the issues that need further investigation and clarification. Recently, special attention has been paid to nitride-based UV emitters. For future applications, UVC (λ: 200–270 nm) emitters are very important (see, e.g., Ref. [127]). This is one of the potential areas requiring high-pressure studies of nitride quantum structures. In particular, the question of efficient doping of UV-C emitters is being studied intensively. This involves the selection of dopants that do not exhibit metastable properties in high Al content AlGaN structures [128]. In contrast to the effective mass-like states, which are highly efficient in supplying current-carrying carriers, the metastable dopants cause the carriers to freeze. The application of pressure is one of the key methods for distinguishing between these types of dopants.

In advanced light emitters, it will be critical to push the limits of GaN-based LEDs for even higher efficiencies and to push light emission into the red color region (more indium in the device QWs) [129]. This will require a better understanding and control of the strain issues resulting from the large lattice mismatch between the different epitaxial layers forming the red emitter.

The application of hydrostatic pressure is very useful for solving the lattice mismatch problem. In the last context, special attention should be given to research on red micro LEDs, which are highly useful in display technologies. In such studies, it is especially important to identify the nature of optical emission and the role played by various factors influencing the overall emission properties of quantum structures. Pressure-dependent experiments and theory can be very helpful for this purpose. In particular, to describe the influence of the effects of internal strain, localization, and internal electric field on the recombination mechanisms, measurements of (i) the Burstein-Moss effect, recombination rates, and their pressure dependence, and (ii) time-resolved high-pressure PL as a function of temperature and excitation power were performed. To estimate the effect of the strain distribution caused by blurred interfaces on the energies and oscillator strengths of the optical transitions, a comparison of polar and nonpolar quantum structures as a function of pressure would be very valuable.

GaN/AlGaN transistors represent a revolution in next-generation power electronics due to their high-power and high-frequency capabilities [130]. Research has focused on improving device performance and reliability and exploring new device architectures, such as GaN-on-Si, for integration with existing chip technologies.

The exploration of other nitride materials, such as BN, for their unique properties, is a growing area. Their heterostructures with GaN and AlGaN offer new functionalities. This research should lead to new applications in high-power electronics, deep UV devices, and quantum technologies. The integration of nitride semiconductors with other material systems such as graphene, various superconductors (e.g., NbN), ferroelectrics, and thermoelectrics (e.g., BaTiO_3_ and SrTiO_3_) to create novel devices is also a hot area. This could lead to new functionalities and improved performance in areas such as solar cells, photodetectors, and optical integrated circuits.

Future developments will require coordinated studies of different specially designed systems, including polar, semipolar, and nonpolar quantum structures with controlled doping, which can be studied experimentally and modeled theoretically. These considerations can be extended to other semiconductor structures that are less studied but have great potential for applications, such as wurtzite or cubic oxide-based systems.

## 6. Summary

This review shows how high pressure helps identify and characterize the properties of semiconducting materials. To illustrate the role of hydrostatic pressure as a research tool in semiconductor physics, III–V nitrides are used as an example. Special emphasis is placed on the effects of high pressure, which plays a crucial role. Beginning with a description of high-pressure techniques in crystal growth processes, trends in the pressure behavior of nitrides are described in the context of typical III–V semiconducting compounds.

It has been shown that a rule that describes the *E_g_* behavior with pressure in III–V semiconductors can be reformulated by including ionicity to better explain the trends in bandgap behavior under pressure. The very small band gap pressure coefficients of nitrides confirm their high ionicity, which is more typical of II–V semiconductors. The effects of the filling of the conduction band under pressure and the pressure dependence of the effective mass have been described using the example of InN, which has one of the smallest band gaps of all semiconductors.

Pressure experiments have revealed remarkable differences in the stability of the crystal lattice under pressure between nitride compounds. Trends in pressure-induced phase transitions were discussed, with particular emphasis on explaining the difference in the phase transition pressure values between GaN and AlN.

The high-pressure technique is an efficient experimental tool for characterizing native defects and impurities in semiconductors. By comparing the experimental and theoretical pressure coefficients, hydrostatic pressure can help identify the origin of different PL spectral lines.

Critical to the application of nitrides in optoelectronics is the identification of the most effective donors and acceptors in these materials that enable *n*- and *p*-type conductivity. Of particular interest are DX centers, which are deep levels strongly coupled to the crystal lattice associated with donors in III–V semiconductors. They are important because of their metastable nature, which allows them to generate both extended and localized states. This review shows that the transition from a shallow hydrogenic state to a highly localized state associated with the DX centers can be most effectively induced by hydrostatic pressure.

The main results and conclusions from the pressure studies of wurtzite QW structures such as GaN/AlN, GaN/AlGaN, GaN/AlInN, and InGaN/GaN with different QW and QB thicknesses and compositions grown along the polar and nonpolar wurtzite directions are presented. It is shown that high-pressure techniques, in particular, spectroscopic studies of the luminescence properties of nitride QWs, have confirmed the dramatic change in these properties under pressure. The experiments also included the dependence on QW geometry, crystallographic orientation, and lattice mismatch between QWs and QBs or QWs and substrates. The experimental data were compared with the calculated electronic properties of the studied structures. An interesting and specific feature of quantum structures in polar wurtzite structures is the presence of a built-in electric field. The related family of effects, very important for optoelectronic applications, is known as the quantum-confined Stark effect. It has been shown that the application of hydrostatic pressure significantly helps to identify the negative role of QCSE and to find effects that lead to the reduction of its “harmful” role in quantum structures.

Special attention has been paid to the role of hydrostatic pressure in the study of short-period nitride superlattices. It was shown that the high-pressure calculations and measurements of the InN/GaN SL bandgap pressure coefficients helped to solve the problem of discrepancies between the experimental and calculated results. Explaining these discrepancies led to the well-documented conclusion that epitaxial growth of high indium In*_x_*Ga_1−*x*_N (*x* > 0.33) SLs is not possible. This highlights the fundamental difficulties in the fabrication of long wavelength InGaN/GaN optoelectronic devices.

In Section 5, it has been shown that hydrostatic pressure can help formulate indications for the design and construction of optoelectronic devices. Using InGaN/GaN LEDs and LDs as an example, it has been shown that the application of hydrostatic pressure significantly helps to identify the negative role of QCSE and to find effects that lead to the reduction of its “harmful” role in optoelectronic devices and to improve the device performance.

Finally, perspectives for future high-pressure studies based on more advanced measurement techniques and more advanced theoretical models were briefly outlined. These include the influence of hydrostatic, tetragonal, and uniaxial strain on polarization effects, identification of the nature of optical emission, determination of the influence of the effects of internal strain, localization, and internal electric field on recombination mechanisms, and the role of various factors that influence the emission properties of different quantum structures. Special attention is paid to nitride-based UV emitters. The experimental techniques and theoretical models used can be extended to other semiconductor systems, such as relatively unexplored wurtzite or cubic oxide structures, which are promising for applications in optoelectronics and photonics.

## Figures and Tables

**Table 1:** Basic parameters of nitrides: band gaps, *E_g_*, pressure coefficient, *dEg*/*dp*, effective lattice constants, *a_eff_*, and ionicities *f_i_*.

	InN	GaN	AlN
*E_g_* (eV)	0.7–0.8 ^a^, 0.76 ^b^, 0.61 ^c^	3.47 ^d^	6.04 ^e^
*dE_g_*/*dp* (meV/GPa)	27.4 ^f^, 29 ± 1 ^g^	41.4 ^h^	49 ^e^
Lattice constant, *a_eff_* (Å)	4.98	4.50	4.37
Ionicity Ref. [34]	0.859	0.770	0.775
Ionicity Ref. [35]	0.578	0.500	0.449
Ionicity Ref. [36]	0.853	0.778	0.794

^a^ Ref. [1] Absorption, PL, and reflectance, 2002. ^b^ Ref. [38] PL, 2002. ^c^ Ref. [39] PL, 2003. ^d^ Ref. [40] Absorption at 20 K, 1991. ^e^ Ref. [41] Absorption, 2002. ^f^ Refs. [5,42], PL, 2007, 2008. ^g^ Ref. [43] Absorption, 2010. ^h^ Ref. [44] PL, 1999.

**Table 2:** Experimental values of the bulk modulus *B* and phase transition pressure *P_T_* for the nitrides considered.

	InN Ref. [46]	GaN Ref. [46]	AlN Ref. [46]
*B* (GPa)	125.5	237	207.9
*P_T_* (GPa)	12.1	52.2	22.9

**Table 3:** Experimental values of InN, GaN, and AlN effective masses m* (in units of *m*_0_).

	InN	GaN	AlN
m*	0.05 ^b^, 0.07 ^c^, 0.044 ^d^	0.20 ^e^ 0.18–0.29 ^f^	0.40 ^e^ 0.29–0.45 ^f^
dm*/*dp*	0.04/GPa ^a^		
dlnm*/*dlnV*		1.1–1.2 ^f^	1.7–1.8 ^f^

^a^ Ref. [48]. ^b^ Ref. [49]. ^c^ Ref. [50]. ^d^ Ref. [51]. ^e^ Ref. [52]. ^f^ Ref. [53].

## Data Availability

No new data were created or analyzed in this study. Data sharing is not applicable to this article.
